# Metagenomic next-generation sequencing for the detection of viruses from respiratory samples: a systematized review on current methods and future directions

**DOI:** 10.1186/s12985-026-03269-0

**Published:** 2026-08-05

**Authors:** Pushpa Kumari, Sudheesh N, Krishna Kumar Ballamoole, Babak Afrough, Anitha Jagadesh

**Affiliations:** 1 Manipal Institute of Virology, Manipal Academy of Higher Education, Manipal, India; 2 Nitte (Deemed to be University), Nitte University Centre for Science Education & Research, Department of Infectious Diseases and Microbial Genomics, Mangalore- 575 018, India, Nitte (Deemed to be University), KS Hegde Medical Academy (KSHEMA), Department of Microbiology, Mangalore- 575 018, India; 3 UK Public Health Rapid Support Team, UK Health Security Agency, and 10 South Colonnade, London E14 4PU, UK

**Keywords:** Respiratory diseases, Respiratory viruses, Metagenomic next-generation sequencing, Amplification strategies, Genome coverage

## Abstract

The comprehensive identification of viruses using metagenomic next-generation sequencing (mNGS) has revolutionized the infectious disease diagnosis. Accurate detection of viruses on time is crucial to effectively control the outbreak. This review has meticulously analyzed the use of mNGS to identify viruses from respiratory samples, emphasizing how sample enrichment and amplification methods affect viral detection efficiency and genome coverage. A total of 66 studies were considered to evaluate sequencing workflows and their alignment with routine molecular techniques. Among these studies, the most frequently used method to enrich viruses was centrifugation (50%), followed by enzymatic treatment (45%). Sequence-independent single-primer amplification (SISPA) and switching mechanism at the 5’ end of RNA template (SMART) was most commonly used amplification methods, which produced better genome coverage for most of the viruses. While mNGS showed better detection capabilities, they showed variable agreement with routine diagnostic methods, with sensitivity varying from 62.5% to 95%. Rhinovirus was detected mainly in lower respiratory tract infections (68%) and respiratory syncytial viruses in upper respiratory tract infections (60%). Although mNGS has the potential, its routine use is hampered by various technical issues. Overcoming these limitations through optimized sequencing protocols and greater cost-effectiveness will be pivotal in integrating mNGS into clinical applications.

**Supplementary Information:**

The online version contains supplementary material available at https://doi.org/10.1186/s12985-026-03269-0.

## Introduction

Every year, respiratory viral infections are responsible for millions of deaths and nearly 80% of all diagnosed cases of acute respiratory infections (ARI), making them a significant global health burden [1–4]. Approximately, 9% of the global population is infected with the influenza virus each year, with 3–5 million severe cases [5–7]. Most commonly detected viruses among ARI patients are influenza viruses, respiratory syncytial viruses (RSV), adenoviruses and rhinoviruses. In addition to these, increased detection of other respiratory viruses like human metapneumovirus (HMPV) observed during early 2025 in China, highlights the dynamic nature of respiratory viral epidemiology [8, 9].

**Fig. 1:**
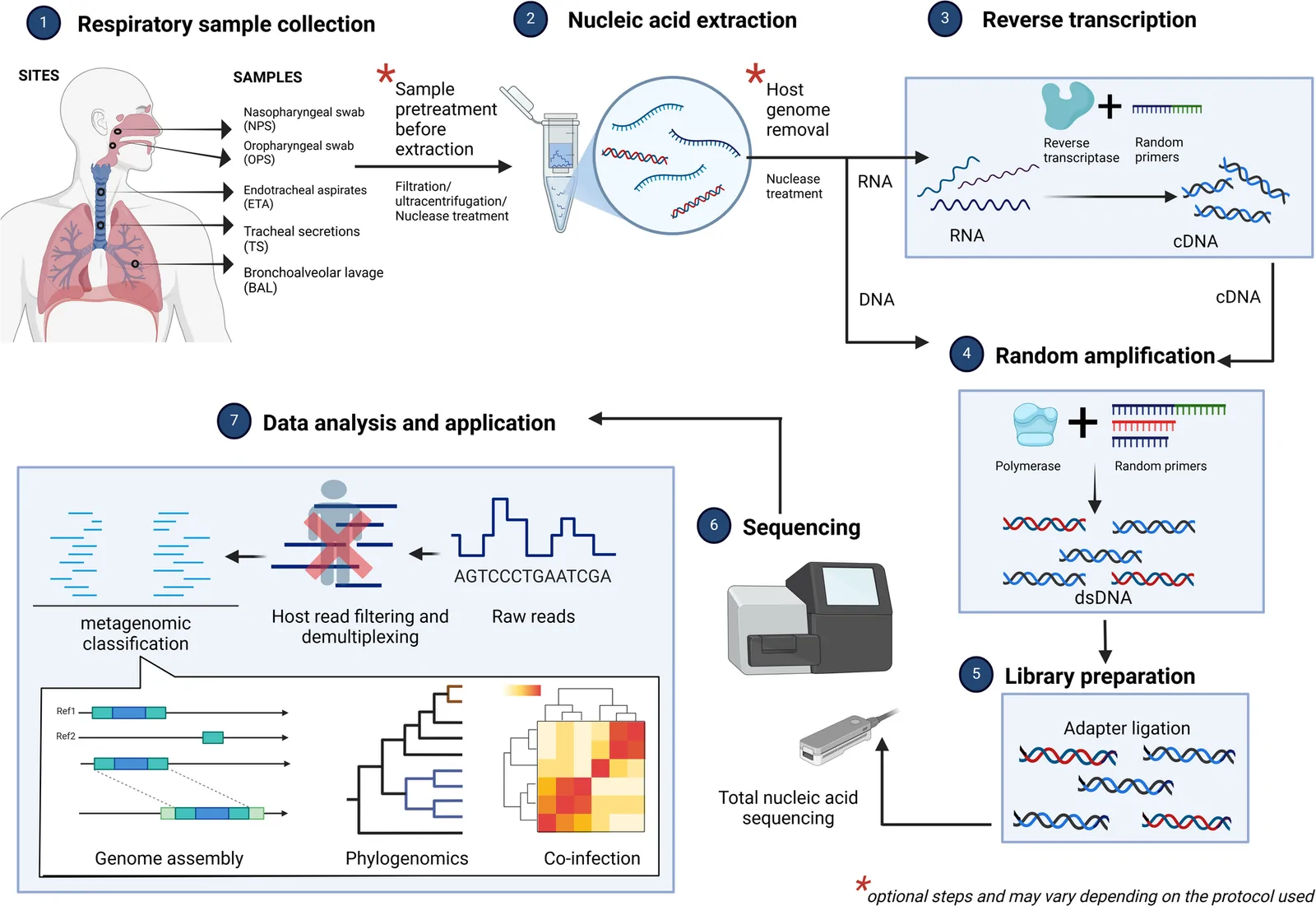
Workflow for untargeted metagenomic sequencing of respiratory samples for viral detection

A significant portion (5–40%) of patients hospitalized with respiratory illnesses do not have an identified infectious agent, even when the viral etiology is suspected [10, 11]. This reflects the diagnostic gap of the currently used routine molecular methods, which has the capability to detect only known viruses and fail to identify divergent and emerging viruses [12]. Consequently, interest towards mNGS has increased in the clinical settings for comprehensive and untargeted diagnostic approaches [13]. Unlike routine molecular methods, it enables pathogen-agnostic detection of a broad spectrum of viruses, including those that are present in low abundance and cannot be cultured [14, 15]. The clinical utility of mNGS has been exhibited in severe and unexplained respiratory illnesses among pediatric patients [16]. The workflow of mNGS for respiratory virus detection, involving the collection of samples, sample enrichment, nucleic acid amplification, sequencing, and bioinformatic analysis, has been illustrated in Fig. 1.

The effectiveness of mNGS in detecting viruses from respiratory samples mainly depends on the sample enrichment method and amplification techniques used, as these samples predominantly contain host and other microbial genomes [17, 18]. Various amplification methods have been developed to ensure low-abundance viruses are also detected, such as sequence-independent single-primer amplification (SISPA) and whole transcriptome amplification (WTA) [19, 20]. Yet, the extensive use of mNGS in clinical settings is hindered by logistical and technical issues, such as high costs, a lack of standardized protocol, and bioinformatic analysis necessary to ensure reproducibility and reliability [21–23].

Detailed information on the applications and advancements of mNGS in the field of infectious diseases has been provided by recent reviews [21, 24–27]. Although the focus on the sample enrichment method and amplification processes used to increase the yield of viral nucleic acid from complex respiratory samples is limited [21, 24–27]. To ensure its successful integration into the global surveillance networks, there is a need to redefine and standardize these preanalytical processes to improve the diagnostic accuracy and turnaround time. To overcome this gap, the objective of our review is to (i) provide an overview of the sample enrichment methods currently used, (ii) outline the amplification methods used in the detection of viruses using untargeted sequencing, and (iii) assess the effectiveness of various amplification techniques in providing comprehensive viral genome coverage.

## Methods

### Study design

The systematized review was carried out following the guidelines outlined by the Preferred Reporting Items for Systematic Reviews and Meta-Analyses (PRISMA) [28]. Ethical approval was not needed, as the study did not involve human or animal subjects or specimens.

### Literature search strategy

A comprehensive literature search was performed in PubMed to identify articles published between January 1, 2011, and November 15, 2024. Boolean operators were applied to refine keyword combinations (Table 1). The initial search strategy was developed for PubMed, and the same search strategy was used for Scopus and Web of Science (*supplementary material*). Reference management and deduplication were carried out using Zotero, followed by a systematic screening of titles and abstracts and full-text review based on eligibility criteria.

**Table 1:** Columns 1,2, and 3 contain search terms that are grouped together using AND/OR

Column 1	Column 2	Column 3
Respiratory viruses	Metagenomic	Clinical samples
RNA virus	Viral sequencing	Respiratory samples
DNA virus	Next-generation sequencing	
	High throughput sequencing	
	Untargeted	

Example search term: (respiratory viruses) AND (untargeted viral sequencing) AND (clinical samples)

### Eligibility criteria

Studies were included if respiratory samples were utilized from patients diagnosed with respiratory diseases or with symptoms of respiratory infection and employed mNGS platforms for viral detection.

Studies were excluded if (i) samples used in the study were environmental or nonrespiratory human samples or cultured clinical isolates, (ii) viruses were detected through targeted viral sequencing, (iii) only abstracts of articles were accessible, (iv) studies not published in English, and v) review articles were excluded. Detailed inclusion and exclusion criteria are mentioned in Table 2.

**Table 2:** Inclusion and exclusion screening criteria

Inclusion criteria	Exclusion criteria
Pertaining to respiratory infections associated with respiratory viruses	Samples from non-human sources (e.g., environmental or animal samples) and non-respiratory human samples (e.g., blood, stool, CSF)
Human respiratory samples	Viral sequencing from cultured clinical isolates or mock viral communities
Untargeted viral sequencing	Viral targeted sequencing using specific primers or probe-based capture
Next-generation sequencing technology was employed	Studies not published in English
Papers published in the search year range of 1 January 2011, to 15 November 2024	Only abstracts of the articles were accessible
Full-text articles	Review articles
Studies available in English language	

## Results

### Identification and inclusion of relevant studies

**Fig. 2:**
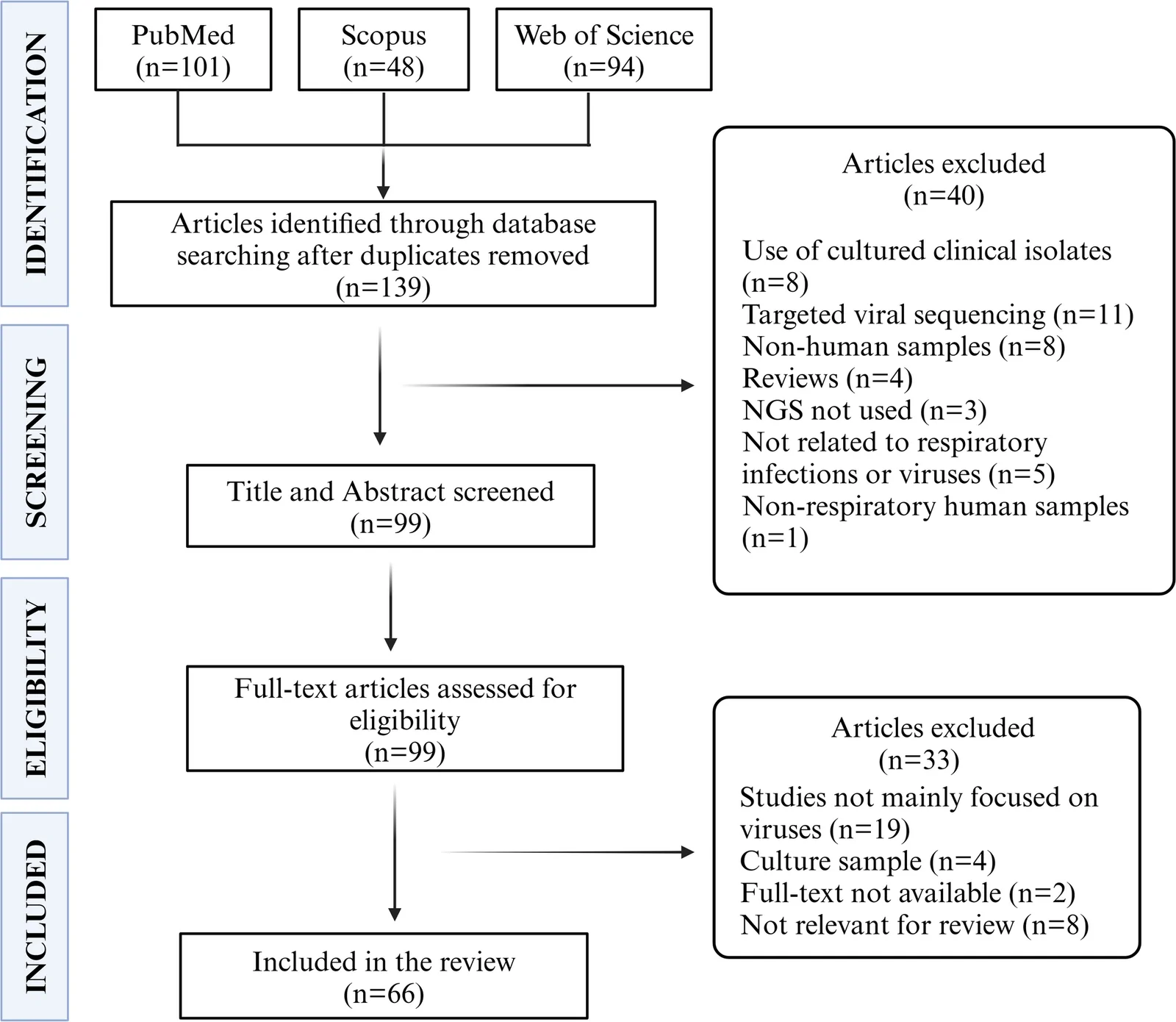
PRISMA flow diagram illustrating the process of literature search and selected articles

A systematic search across three databases yielded a total of 243 articles (Fig. 2). Excluding duplicates, 139 unique records were screened for title and abstract. Ninety-nine studies were selected for full-text evaluation. Out of 99, 33 articles were excluded for not meeting the predefined inclusion criteria, and consequently, 66 studies were eligible and included. A summary of these studies is available in *Table S1 (Supplementary Material).*

### Characteristics of selected studies

The selected studies were conducted across various geographical regions, with Europe contributing to the highest proportion (36%, *n* = 24), followed by Asia (29%, *n* = 19), North America (21%, *n* = 14), and South America (8%, *n* = 5). Fewer studies also originated from Africa (4%, *n* = 3) and Australia (2%, *n* = 1). Over time, the publication output varied, reaching peak in 2020 (16.6%), probably as a result of increased research focus on the COVID-19 pandemic. In other years, publications ranged from 1.5% (*n* = 1) to 12% (*n* = 7), reflecting steady but fluctuating research activity in this field *(supplementary Fig. 1)*. Regarding populations, 39% of the studies (*n* = 26) focused on pediatric patients, 16% (*n* = 11) on adults, 10% (*n* = 7) on mixed groups, and 34% (*n* = 22) did not specify. In terms of respiratory infections, 15% (*n* = 10) included upper respiratory tract infections (URTIs), 33% (*n* = 22) included lower respiratory tract infections (LRTIs), 21% (*n* = 14) included both URTIs and LRTIs, 5% (*n* = 3) included targeted lung transplant recipients, and 26% (*n* = 17) did not specify the respiratory infection type. The Illumina sequencing platform was predominantly used (76%, *n* = 50), followed by Oxford Nanopore technologies (15%, *n* = 10), Ion Torrent (4%, *n* = 3), Roche (4%, *n* = 3), and Pacific Biosciences (2%, *n* = 1). Key characteristics are summarized in Table 3.

**Table 3:** Characteristics of selected studies (*n* = 66)

Characteristics	*N* (%)
**Location of the study**	
Europe	24 (36%)
Asia	19 (29%)
North America	14 (21%)
South America	5 (8%)
Africa	3 (4%)
Australia	1 (2%)
**Study population**	
Children	26 (39%)
Adults	11 (16%)
Mixed	7 (10%)
Not reported	22 (34%)
**Study on types of respiratory infections** ^**a**^	
Upper Respiratory Tract Infections (URTI)	10 (15%)
Lower Respiratory Tract Infections (LRTI) Mixed (Both URTI & LRTI)	22 (33%) 14 (21%)
Lung transplant recipients	3 (5%)
Not reported	17 (26%)
**Specimen type**	
Upper respiratory samples (NPS, OPS, throat swab, NPA, NPW, OPW)	46 (69%)
Lower respiratory samples (BAL, ETA, sputum, tracheal aspirates)	3 (5%)
Both upper and lower respiratory samples	17 (26%)
**NGS platform** ^**b**^	
Roche	3 (4%)
Ion Torrent	3 (4%)
Illumina	50 (76%)
Pacific BioSciences	1 (2%)
Oxford Nanopore Technologies	10 (15%)

^a^Note:

**Upper Respiratory Tract Infection** includes ARI patients with mild to moderate respiratory symptoms without hospitalization

**Lower Respiratory Tract Infection** includes SARI, COPD, pneumonia, severe respiratory illness, and RTI patients with hospitalization

**Mixed** includes studies that enrolled both mild to moderate and severe respiratory infection patients

^b^Few of the included studies employed one or more NGS platforms

### Sample enrichment methods

Out of 66 studies, 81.8% (*n* = 54) performed sample enrichment, while 18.2% (*n* = 12) did not. Among the studies employing enrichment, centrifugation was the most frequently used method (*n* = 33), followed by enzymatic treatment prior to extraction (*n* = 30), filtration (*n* = 26), enzymatic treatment postextraction (*n* = 15), rRNA depletion (*n* = 7), ultracentrifugation (*n* = 5), and sample concentration (*n* = 6). Most studies applied more than one enrichment method in combination to enhance viral detection sensitivity. Centrifugation and filtration were primarily used to remove cellular debris, with centrifugation speeds ranging from 1500 × g to 16,000 × g and filtration pore sizes ranging from 0.22 to 0.8 μm. Enzymatic treatments involving DNase alone or in combination with RNase are commonly performed to eliminate the host genome [29]. Ultracentrifugation or concentrator-based methods, including the Zymo Research Clean (Zymo Research^®^) and Amicon (Millipore^®^) centrifugal filters, were employed to increase viral concentration [11, 16, 30–33]. The combinations of sample enrichment methods utilized are summarized in Table 4.

**Table 4:** Sample enrichment methods used

Characteristics	*N* (%)
**Sample enrichment methods used**	**54 (81.8)**
**A. Only one method**	**16 (24)**
i. Centrifugation	4 (6)
ii. Enzymatic treatment (pre-extraction)	6 (9)
iii. Enzymatic treatment (post-extraction)	5 (7.5)
iv. rRNA depletion	1 (1.5)
**B. Combination of two methods**	**16 (24)**
i. Centrifugation and filtration	4 (6)
ii. Centrifugation and enzymatic treatment (pre-extraction)	4 (6)
iii. Centrifugation and enzymatic treatment (post-extraction)	1 (1.5)
iv. Centrifugation and rRNA depletion	1 (1.5)
v. Filtration and ultracentrifugation	1 (1.5)
vi. Filtration and enzymatic treatment (pre-extraction)	3 (4.5)
vii. Enzymatic treatment (post-extraction) and concentration	1 (1.5)
viii. Enzymatic treatment (post-extraction) and rRNA depletion	1 (1.5)
**C. Combination of three methods**	**18 (27)**
i. Centrifugation, filtration and concentration	1 (1.5)
ii. Centrifugation, Filtration and Enzymatic treatment (pre-extraction)	9 (13.6)
iii. Centrifugation, Concentration and Enzymatic treatment (post-extraction)	1 (1.5)
iv. Centrifugation, enzymatic treatment (pre-extraction) and rRNA depletion	1 (1.5)
v. Centrifugation, Enzymatic treatment (post-extraction) and Concentration	3 (4.5)
vi. Filtration, Concentration and Enzymatic treatment (pre-extraction)	1 (1.5)
vii. Filtration, ultracentrifugation and enzymatic treatment (pre-extraction)	2 (3)
**D. Combination of four methods**	**1 (1.5)**
i. Centrifugation, filtration, ultracentrifugation and enzymatic treatment (pre-extraction)	1 (1.5)
**E. Combination of five methods**	**3 (4.5)**
i. Centrifugation, filtration, enzymatic treatment (pre-extraction), enzymatic treatment (post-extraction), and rRNA depletion	3 (4.5)
**No sample enrichment methods used**	**12 (18.2)**

### Amplification methods

The in-house method was used for the amplification of nucleic acids in 50% (*n* = 33) of studies. Among the in-house methods, 44% (*n* = 29) employed SISPA, 4% (*n* = 3) employed SMART amplification, and 2% (*n* = 1) employed VIDISCA. Commercial kit-based amplification was used in 48% (*n* = 32) of the studies, demonstrating their growing dominance in the untargeted detection of viruses. No amplification was used in 2% (*n* = 1) of studies, and a small proportion of studies (2%, *n* = 1) did not clearly specify the amplification strategy (Table 5). Given the diversity of amplification strategies, this review focuses on commonly used methods in untargeted viral metagenomic sequencing of respiratory samples, specifically SISPA, SMART, MDA, and dUTP-based strand-specific amplification.

**Table 5:** Amplification methods used

Characteristics	*N* (%)
Amplification methods	
**A. In-house**	**33 (50)**
i. Sequence-Independent, Single-primer Amplification (SISPA) a) *SISPA-6 N* b) *SISPA-8 N* c) *SISPA-9 N*	29 (44) *11 (17)* *11 (17)* *7 (10)*
ii. Switching Mechanism At the 5’ end of RNA Template (SMART) a) *SMART-6 N* b) *SMART-9 N*	3 (4.5) *1 (1.5)* *2 (3)*
iii. Virus discovery cDNA-amplified fragment length polymorphism (VIDISCA)	1 (1.5)
**B. Commercial Kit** ^**c**^	**32 (48)**
i. Switching Mechanism At the 5’ end of RNA Template (SMART)	5 (8)
ii. Whole transcriptome amplification (WTA)	4 (6)
iii. Multiple Displacement Amplification (MDA)	11 (16)
iv. Poly A capture-based random amplification	1 (2)
v. Single-Primer Isothermal Amplification (SPIA)	3 (4)
vi. Random priming-based whole transcriptome amplification	1 (2)
vii. dUTP-based strand-specific amplification	6 (9)
viii. Strand-switching random amplification	1 (2)
**No amplification method used**	**2 (3)**
**Unclear**	**1 (2)**

^c^Two of the included studies used more than one amplification method

#### In-house method

##### Sequence-Independent, Single-Primer Amplification (SISPA)

**Fig. 3:**
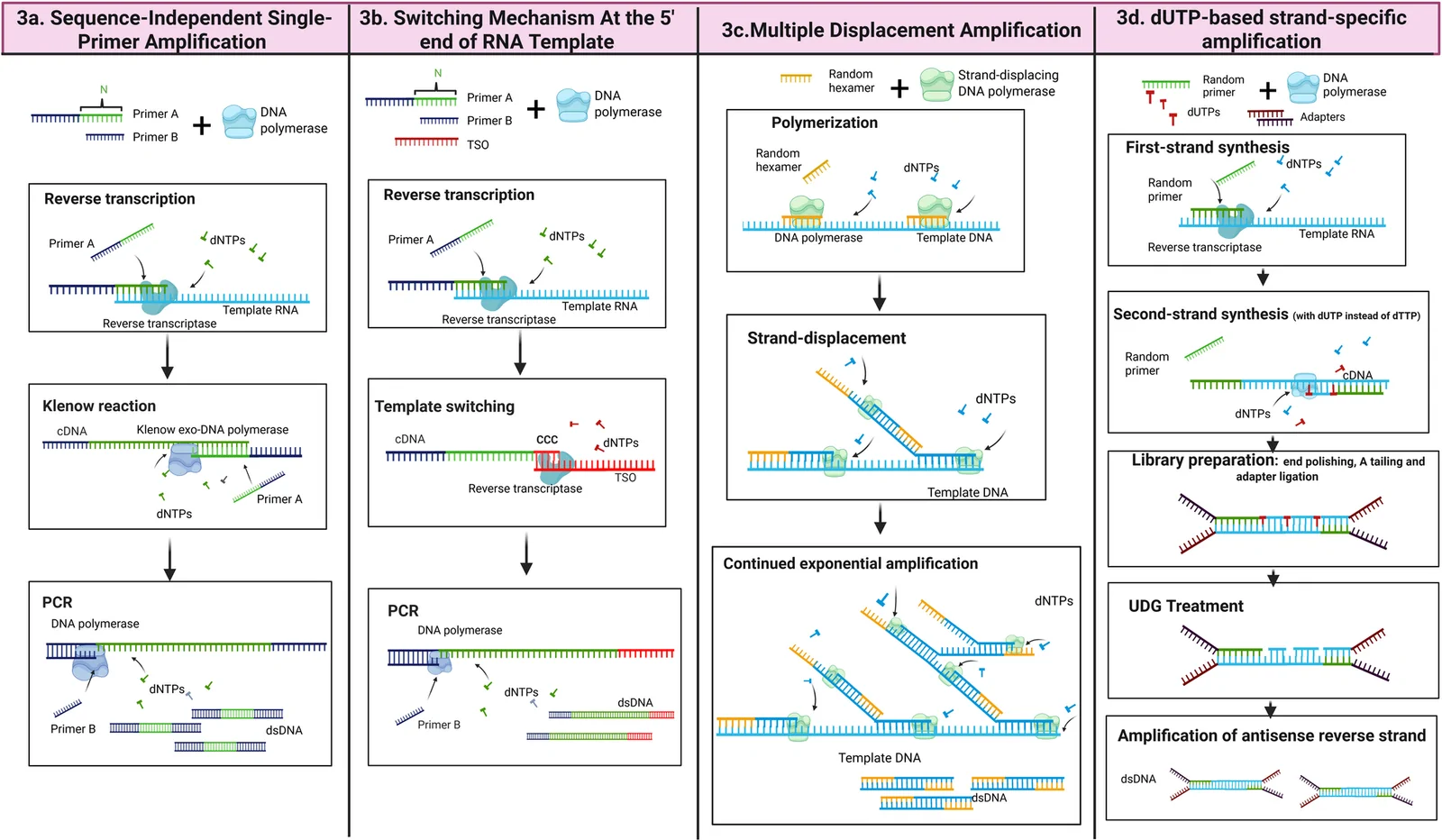
Amplification methods used in the untargeted viral sequencing − 3a. Sequence-Independent Single-Primer Amplification (SISPA), 3b. Switching Mechanism At the 5’ end of RNA Template (SMART), 3c. Multiple Displacement Amplification (MDA), 3d. dUTP based strand-specific amplification

SISPA employs random primers to synthesize complementary DNA (cDNA) from viral RNA or DNA, followed by second-strand synthesis using Klenow exo-DNA polymerase to generate double-stranded DNA (dsDNA) for sequencing (Fig. 3a). The primers are typically designed with a random sequence at the 3’ end (e.g. 6 N, 8 N, or 9 N) and a universal adapter sequence at the 5’ end for subsequent amplification [34]. These primers allow for nonspecific amplification of diverse viral genomes, making SISPA particularly effective in metagenomic applications [35].

##### Switching mechanism At the 5’ end of RNA Template (SMART)

SMART is based on the template-switching activity of reverse transcriptase, enabling efficient synthesis of full-length cDNA from RNA templates [36–38]. During reverse transcription, non-templated cytosine residues are added to the 5′ end of the newly synthesized cDNA. A SMART oligonucleotide, also known as template switching oligo (TSO) with guanine-rich sequence, anneals to these residues, enabling strand switching and continued cDNA synthesis (Fig. 3b). This mechanism preserves strand specificity, improves transcript coverage, and reduces amplification biases, making SMART suitable for metagenomic applications. Over time, SMART amplification has been modified to improve viral genome recovery, evolving from poly(dT) tailing and priming for selective viral RNA capture [38, 39] to primers incorporating specific degenerate 6-nucleotide sequences for broader genome coverage [40]. To further increase the sequencing sensitivity, SMART-9 N primers containing nine degenerate nucleotide sequences have been recently employed [41].

#### Commercial kit-based

##### Multiple Displacement Amplification (MDA)

MDA is an isothermal-based DNA amplification method, commonly used to improve the identification of low-abundance viral genomes in respiratory samples [42]. The process begins with random hexamers annealing to denatured dsDNA and initiating strand synthesis using Phi29 DNA polymerase, characterized by its strong displacement, high processivity, and 3’→5’ exonuclease proofreading activity. By continuously synthesizing complementary strands and displacing previously synthesized DNA strands (Fig. 3c), it enables exponential amplification that generates high molecular weight DNA with uniform coverage [43, 44].

##### dUTP-based strand-specific amplification

It is a method designed to accurately characterize viral transcriptomes in metagenomic sequencing by retaining the strand orientation of RNA during cDNA synthesis. Random or oligo(dT) primers are used to produce cDNA from viral RNA, followed by second-strand synthesis, where dUTP is added instead of dTTP to produce dsDNA molecules with uracil residues. These dsDNA molecules are treated with uracil-DNA glycosylase (UDG) to selectively degrade the strand containing dUTP, leaving only the first-strand cDNA for library preparation (Fig. 3d). These enable the precise differentiation between sense and antisense viral RNA strands and preserve strand specificity. This method also reduces the amplification bias by preventing the loss of strand information and improves metagenomic sequencing efficiency [45].

### Detection of respiratory viruses using untargeted sequencing

**Fig. 4:**
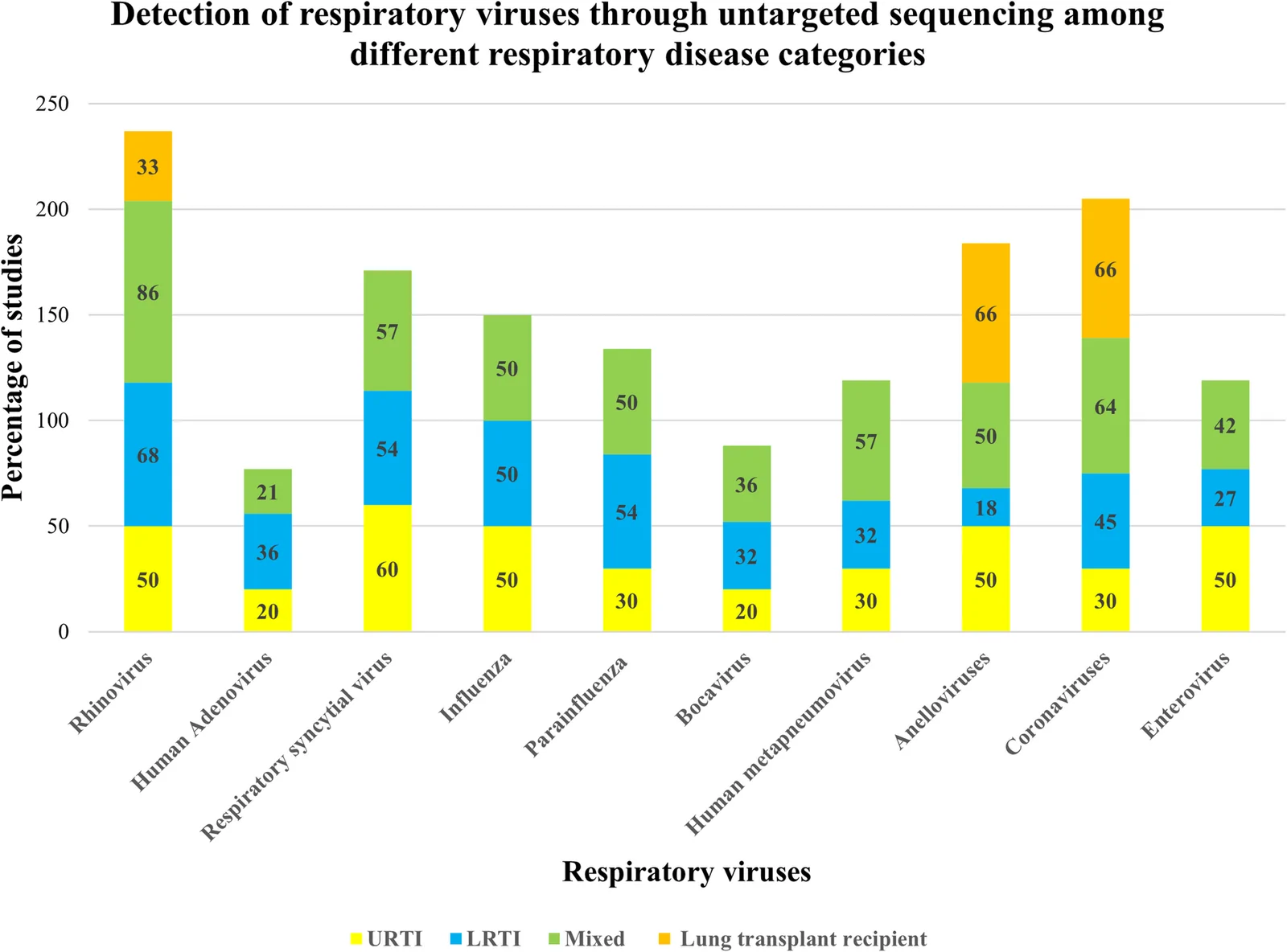
The Graph represents the percentage of studies that reported the detection of respiratory viruses among different respiratory diseases. A bar indicates the total percentage of studies that detected particular respiratory virus

Out of 66 studies, rhinovirus was detected in 62% (*n* = 41), followed by coronavirus (59%, *n* = 39), influenza virus (47%, *n* = 31), RSV (46%, *n* = 30), parainfluenza virus (42%, *n* = 28), HMPV (38%, *n* = 25), enterovirus (30%, *n* = 20), anellovirus (27%, *n* = 18), adenovirus (26%, *n* = 17), and bocavirus (26%, *n* = 17). Respiratory virus detection across different respiratory disease categories is illustrated in Fig. 4.

Following the overall detection frequencies, virus-specific distribution patterns were evident across clinical syndromes. Rhinovirus genotypes A, B, and C were detected in URTI and LRTI, with genotype A predominating in URTI [46–50] and genotypes A and C in LRTI [31, 33, 42, 51–56]. Untargeted sequencing also revealed novel and highly divergent strains of rhinoviruses [31, 33, 57] and clinical severity associations [56, 58]. The detection of coronaviruses in the URTI was reported in only 3 studies, primarily HCoV-229E, NL63, and OC43 being the most frequently identified types [10, 47, 48], whereas LRTI studies showed coronavirus OC43 predominance [59] and enabled metagenomic identification of the novel coronavirus 2019-nCoV from BALF [60]. Influenza A, B, and C were detected in URTI [10, 47, 48, 61] and LRTI [16, 31, 58, 62–65], with influenza A H3N2 and B predominating in URTI [10, 47, 48, 61]. Studies have also identified genetic mutations, intra-subtype reassortment, and antiviral resistance-associated genotypes [66, 67], while most strains remain susceptible to neuraminidase inhibitors [57, 65, 67].

Untargeted mNGS also enabled the simultaneous detection of diverse respiratory viruses [16, 31, 42, 46, 68–70] and non-respiratory viruses, such as human papillomavirus [30, 46, 49, 51, 69, 71–76], human herpes viruses [49, 50, 53, 61, 66, 69, 71–75, 77–83], and polyomaviruses [49]. Studies have also revealed anelloviruses, abundance in immunocompromised populations [70, 71], traced nosocomial transmission [11, 84, 85], and facilitated the discovery of novel respiratory-associated viruses such as human respiratory-associated PSCV-5-like virus (HRAPLV) and scaffold viruses from clinical samples [72, 86].

### Bioinformatic pipelines used in the selected studies

Of 66 studies, 15.1% (*n* = 10) used commercially available bioinformatic platforms such as CodonCode Aligner software [10], CLC Genomic workbench [16, 33, 51, 86], Geneious v9.1 software [29, 85, 87], and Genome detective application [18, 42]. Most of these were validated to analyze sequences from clinical samples [16, 18, 33, 42, 51, 86, 87]. Another 83.3% (*n* = 55) of studies used open source bioinformatic tools such as FastQC, Trimmomatic, and Cutadapt to assess the quality of reads and trim adapters and low-quality bases. Bowtie2, BWA, SOAP Aligner, or Minimap2 were used to remove host reads by mapping reads to the human reference genome (hg18, hg19, or GRCh38). Several pipelines also removed bacterial, fungal, ribosomal RNA, and mitochondrial sequences using reference databases such as SILVA, RefSeq bacteria, or Human Microbiome Project genomes. SPAdes, MEGAHIT, Velvet, and SOAPdenovo2 were used for de novo assembly to improve viral genome recovery and confirm taxonomic hits. Assembled contigs were screened against nt/nr databases using BLAST. Phylogenetic analyses were performed using MAFFT, ClustalX, MEGA, and FastTree. A few open-source bioinformatic pipelines have also been validated, such as the BIOPET Gears pipeline [65], ID-seq pipeline [30], ChanZuckerberg ID (CZID)portal [56], PATHDET metagenomic pipeline [88], BugSeq cloud-based analysis portal [76], and SURPI+ computational pipeline [58].

### Does the type of amplification method influence the detection and genome coverage of the virus?

A comparative evaluation of amplification methods used in untargeted mNGS reveals notable differences in their performance in viral genome recovery and detection efficiency across respiratory samples with varying Ct values (Table 6). Among the in-house methods, SISPA protocols, specifically SISPA-6 N, 8 N, and 9 N, were employed across a wide range of Ct value range (15 to 44) and enabled the identification of a diverse panel of respiratory viruses, including SARS-CoV-2, influenza, bocavirus, rhinoviruses, enteroviruses, and anelloviruses [17, 32, 47, 48, 64, 67, 89–91]. SISPA-8 N, in particular, demonstrated consistently high genome coverage (51.72% − 100%), suggesting superior performance in low viral load samples [17]. Importantly, several viruses undetected by routine diagnostics were identified using SISPA, highlighting the enhanced sensitivity of this approach [11, 30, 46, 56, 72]. SMART-based amplification, including SMART-6 N and SMART-9 N, was assessed in samples with Ct values from 19 to 33. These methods also provided high genome coverage (2.03% − 99.92%) and were capable of detecting both RNA and DNA viruses [40, 41, 60]. Among commercial kits, MDA and dUTP-based strand-specific amplification demonstrated broad applicability across Ct values ranging from 10 to 40 and 11 to 38, respectively [18, 29, 42, 54, 63, 65, 87, 92, 93]. Both methods detected a wide range of viruses but exhibited considerable variability in genome coverage (0.13% − 100%) compared to the SISPA method [42].

**Table 6:** Comparison of amplification methods for the detection and genome coverage of viruses

Amplification methods ^d^	Ct value of samples	Virus detected	Genome coverage (%)
**A. In-house**			
i. Sequence-Independent, Single-Primer Amplification (SISPA)			
a) *SISPA-6 N*	17 to 40	SARS-CoV-2, RSV, enterovirus D68, human rhinovirus B, C, influenza A virus, human coronavirus-OC43, anellovirus	0.8–100
	Negative samples	Human bocavirus, enterovirus A and B, anelloviruses, parvoviruses	82–100
b) *SISPA-8 N*	17 to 44	Human rhinovirus, HMPV, parainfluenza 1,2,3, rhinovirus	51.72–100
	Negative samples	HMPV	98
c) *SISPA-9 N*	15 to 39	Influenza virus, HMPV, coronavirus, SARS-CoV-2, enterovirus B, C, bocavirus, rhinovirus, anellovirus	3.63–100
	Negative samples	RSV, human coronavirus-OC43, rhinovirus A, B, C, bocavirus, anellovirus, scaffold virus	0.13–99.81
ii. Switching Mechanism At the 5’ end of RNA Template (SMART)			
a) *SMART-6 N*	19 to 33	Influenza virus, rhinovirus, adenovirus B1, coronavirus,	2.03–98.75
b) *SMART-9 N*	22 to 33	SARS-CoV-2	95.90–99.92
**B. Commercial Kit**			
i. Multiple Displacement Amplification (MDA)	10 to 40	Bocavirus, anellovirus, rhinovirus C, enterovirus, HMPV, RSV, influenza, SARS-CoV-2, parainfluenza virus-3	0.13–100
ii. dUTP-based strand-specific amplification	11 to 38	Influenza A virus, human adenovirus, human coronavirus, human parainfluenza virus-3,4, rhinovirus, SARS-CoV-2, RSV, bocavirus, enterovirus	3–100

^d^Note: Studies that reported Ct values of the samples (if positive), viruses detected and genome coverage of the viruses were only included for the analysis

### Is there agreement between untargeted sequencing and molecular methods currently used for diagnosing viral respiratory infections?

Among 66 studies, 84.8% (*n* = 56) employed routine molecular diagnostic methods for viral detection, while a smaller fraction (15.2%, *n* = 10) relied solely on mNGS. The comparison between routine diagnostics and mNGS revealed variable concordance, largely influenced by factors such as viral genome characteristics, sequencing depth, and bioinformatics workflows [10, 47, 50, 62, 65].

Studies that applied mNGS exclusively for samples tested positive by routine diagnostics reported high levels of agreement [10, 40, 41, 47, 57, 58, 77], i.e., mNGS demonstrated 81.8% agreement with qPCR detecting all high-viral load samples [40] but failed to identify some low-viral load samples with Ct values ranging from 28.03–36.07 [10, 40, 47].

In contrast, studies incorporating both PCR-positive and PCR-negative samples demonstrated variable concordance [16, 52, 54]. Sensitivity variations were observed depending on the viral genome; for example, mNGS exhibited higher sensitivity than qPCR for detecting influenza A (H1N1), PIV-1 and BoV-2. However, its sensitivity for adenovirus was lower [52]. In another study, mNGS detected all viruses detected by PCR and additionally identified other viruses, such as human herpesvirus 5 and enteroviruses [77].

Some studies analyzed mNGS performance on samples that tested negative by routine diagnostics, aiming to determine its utility in detecting missed infections [11, 30, 33, 49, 51, 56, 61, 69, 72–74, 82, 83, 94]. mNGS successfully detected RSV and human coronaviruses in samples previously reported as negative [72]. In another study, viral sequences were identified in 30.5% of PCR-negative samples [82]. Similarly, analysis of samples initially negative by routine RSV immunofluorescence assays revealed the presence of parainfluenza virus 4 and HMPV in a subset of cases [11]. Sensitivity varied by different viruses, with mNGS achieving 62.5 to 95% sensitivity compared to routine methods [32, 40, 47, 50, 54, 57, 58, 62, 65, 67, 76, 84, 91, 93]. The positive predictive value of mNGS remained high in these studies, but the negative predictive value varied depending on sequencing depth and bioinformatics pipelines [54].

### Validation of mNGS results

Validation of mNGS plays a crucial role in ensuring the reliability and accuracy of viral identification in clinical diagnostics. Among studies, 31.8% (*n* = 21) incorporated additional validation methods. Real-time PCR and qPCR were the most commonly used methods in 21.2% (*n* = 14) of the studies to validate the presence of identified viruses [33, 47–50, 57, 62, 69, 74, 75, 77, 86, 90, 94]. Additionally, 9% (*n* = 6) of studies validated findings using an alternative NGS platform [17, 65, 67, 84, 88]. 3% (*n* = 2) of studies employed both real-time PCR and sequencing [11, 71]. An alternative NGS platform was used for cross-validation to verify viral sequences and facilitate complete viral genome assembly.

## Discussion

We systematically reviewed 66 studies that employed mNGS to detect respiratory viruses directly from clinical samples, providing an overview of current methodological practices in this field. Among these studies, different sequencing technologies are utilized to identify respiratory viruses such as Illumina, which uses sequencing-by-synthesis method where optical readout of incorporated fluorescent nucleotides coupled to a reversible terminator is measured; Ion Torrent detects pH changes associated with nucleotide incorporation; Roche 454 relies on pyrosequencing through light detection; Oxford Nanopore measures disruptions in ionic current as nucleotide passes through nanopores; and Pacific Biosciences uses single molecule real-time sequencing in which fluorescently labeled nucleotides are detected in real-time within zero-mode waveguides (ZMVs) during DNA synthesis by a single DNA polymerase [95, 96]. We found that Illumina sequencing platform was most frequently employed in these studies, which is in line with the results from previous study [21]. Prior reviews also discussed the use of both targeted and untargeted NGS techniques, focusing on sequencing platforms and their diagnostic use. The current review builds upon this work by focusing mainly on untargeted mNGS and how amplification strategies affect detection efficiency and genome coverage of viruses.

The main finding of this review is that, despite differences in the results between routine molecular diagnostics and mNGS, they played complementary roles in the detection of respiratory viruses. PCR offered high sensitivity and specificity for known viruses, while mNGS enabled unbiased and agnostic detection of viruses that were not anticipated based on clinical presentations. Several studies reported successful identification of viruses using mNGS, including coinfections and novel strains that were missed by routine molecular techniques, highlighting mNGS’s potential to fill diagnostic gaps in undiagnosed or complex respiratory illnesses. Nevertheless, the effectiveness of mNGS significantly varied by multiple preanalytical factors across the studies. When compared to the single-step methods, studies using combinations of enrichment methods reported better viral reads and genome coverage. A combination of centrifugation, filtration, concentration, and enzymatic treatment was shown to increase viral reads by 15 times and improve the genome for HCoV-OC43 from 4 to 34% to 99.8% [59]. Higher sensitivity and comprehensive viral genome coverage were consistently obtained by combining pretreatment with optimized extraction techniques, like automated or magnetic bead-based extractions [59, 89]. These findings demonstrate the importance of combining several enrichment methods to enhance viral nucleic acids, particularly in respiratory samples that have a significant amount of host derived material. The selection of amplification strategies also has a crucial impact on the sensitivity of detecting viruses and their genome coverage, which has been indicated across studies. For example, SISPA (6 N, 8 N, or 9 N) has consistently achieved high genome coverage (> 90%), making it reliable for the detection of respiratory viruses [17, 67]. Similar to this, SMART performed exceptionally well showing high genome coverage, especially for RNA viruses [77]. MDA on the other hand demonstrated varying genome coverage (0.13–99.23%), despite being effective for small and circular genomes, underscoring its limitations in mixed viral populations [71, 93]. These findings indicate that amplification induced bias is still a significant drawback of untargeted metagenomic sequencing, requiring the choice of methods according to the viral load, genotype and analytical goals.

In addition to the methodological performance, the spectrum of respiratory viruses reported here might not accurately represent true epidemiology. While rhinovirus was the most commonly detected virus, many studies only examined prescreened negative samples, a mix of positive and negative samples or only positive samples, which may introduce selection bias and limit generalizability to larger clinical populations. Another significant source of variation in the untargeted detection of respiratory viruses was bioinformatic analysis. Even though processes like genome quality check, host-read removal, and taxonomic classification were essentially the same, there was significant variation in tool selection, reference database, and reporting thresholds. Although the dominance of open-source tools stemmed from their accessibility and flexibility, cross-study comparison and reproducibility were hampered by inconsistent parameter settings and reporting thresholds. Validated pipelines increased clinical interpretability, but they were still underutilized.

A significant limitation of this review is that the assessment of the amplification method for the effectiveness in providing complete genome coverage is limited by the fact that many studies did not provide genome coverage for particular amplification techniques. Also, the present study is a systematized review rather than a formal systematic review, a structured risk of bias assessment was not performed. In addition, a meta-analysis was not undertaken because of considerable methodological heterogeneity in the included studies such as sequencing platforms other than Illumina were each represented by only one to three studies, while sample enrichment strategies varied substantially, with several individual methods or combinations of methods used in a limited number of studies. Clinical heterogeneity was also evident, as only a few studies included patients with specific respiratory infection types, and respiratory sample types varied across studies, with lower respiratory tract specimens represented in only three studies. These methodological differences, together with the diversity in bioinformatic workflows, limited the comparability of study findings and quantative synthesis. In spite of this, we aimed to provide a comprehensive and accurate analysis of mNGS in detecting respiratory viruses.

## Challenges and future directions

Untargeted viral sequencing for clinical diagnostics presents challenges that are multifaceted. (i) Sensitivity remains a major barrier. VIDISCA detected viruses only at ≥ 10^6^ copies/mL, whereas rapid SMART-9 N and dUTP-based methods achieved lower limits (10^3^ − 10^4^ and 543 viral copies/mL). However, sensitivity remains method dependent, limiting comparability across studies [10, 41, 76]. (ii) Technical limitations, such as the absence of proper controls and high background contamination from laboratory reagents, such as silica-based spin-filter columns, introduce false-positive results complicating metagenomic approaches [77, 93]. (iii) Lack of healthy control samples in various studies limits the pathogenic significance of detected viruses [31, 72, 83, 88]. (iv) Amplification bias from short hexamers used in Endoh-tagged primers has resulted in incomplete genome coverage [97]. (v) Bioinformatics and workflow standardization remain key obstacles, and the absence of consensus criteria for defining a positive viral detection for low-abundance or clinically uncertain viruses [42, 57, 58, 66, 76, 84]. (vi) Cost and turnaround time [56–58, 62, 66, 67, 76]. mNGS costs ~ £140 per sample while multiplexing, versus £56 per sample for routine influenza A/B and RSV testing using GeneXpert, highlighting economic barriers to broader mNGS application [67]. Beyond reagents’ cost, mNGS limits scalability and timely clinical decision-making, especially in resource-limited settings, where it requires substantial infrastructure, computational resources, data storage, skilled personnel, and longer turnaround time. (vii) Host nucleic acid contamination is another major problem, limiting viral read percentage and requires multiple depletion steps that increase processing time and cost [41, 53, 63, 67, 76, 77].

There is a need for a harmonized workflow to overcome these challenges. Future studies should focus on using appropriate enrichment methods to maximize the sensitivity of mNGS. In clinical settings where rapid pathogen identification is important, techniques such as adaptive sampling provide a promising approach to selectively enrich viral sequences by dynamically rejecting host DNA [98]. Moreover, maximizing viral genome coverage is possible by addressing amplification biases linked to short random hexamers, like those in tagged Endoh primers [97]. According to the studies that have assessed various amplification techniques, random priming with N10 or N12 primers produced better genome coverage [99]. These results suggest that primer length optimization and careful selection of amplification methods could improve the accuracy and comprehensiveness of viral sequencing. In order to minimize sample-related confounding factors and enable robust assessment of sensitivity, viral diversity and genome coverage, future research should include a variety of sample matrices and compare key methodological steps in a systematic manner. Additionally, to enhance accuracy and its application in clinical practice, streamlined bioinformatic pipelines with improved reference databases and more efficient sequencing chemistries are required [10, 41, 50]. There is also a need for cost-benefit analysis and clinical validation studies [58]. Given its capacity to detect both expected and unexpected viruses, mNGS has the potential to bridge diagnostic gaps in cases with negative routine testing or atypical presentations, facilitating well-informed clinical decision-making and improving patient care and outcomes.

## Conclusion

Our review highlights the broad application of metagenomic sequencing to detect known and divergent as well as novel viruses from respiratory samples and also to reveal coinfection conditions. The findings of mNGS were also confirmed in many cases using routine molecular methods and additional sequencing platforms, which might impact the clinical outcomes. However, its implementation as a routine diagnostic method is difficult at present, due to many technical and logistical issues. Through this review, we found that amplification methods such as SISPA and SMART often provide a high viral genome (> 90%) for many viruses, but MDA was better suited for small and circular genomes. This implies that to ensure optimal performance of the sequencing workflow, the amplification method selected should correspond to the anticipated viral targets and the required genome coverage. Ongoing research on reducing host genome, standardizing metagenomic sequencing workflows and improving sequencing chemistries will be essential to increase accuracy and reduce turnaround time, and thereby ensuring effective integration of the mNGS method into clinical applications.

## Supplementary Information

Supplementary Material 1

## Data Availability

No datasets were generated or analysed during the current study.

## Abbreviations

BAL: Bronchoalveolar Lavage
CMV: Cytomegalovirus
COPD: Chronic Obstructive Pulmonary Disease
CRISPR: Clustered Regularly Interspaced Short Palindromic Repeats
dUTP: Deoxyuridine Triphosphate
ETA: Endotracheal Aspirate
HPIV: Human Parainfluenza Virus
HPV: Human Papillomavirus
LRTI: Lower Respiratory Tract Infection
MDA: Multiple Displacement Amplification
mNGS: Metagenomic Next–Generation Sequencing
NGS: Next–Generation Sequencing
NPA: Nasopharyngeal Aspirate
NPW: Nasopharyngeal Wash
OPW: Oropharyngeal Wash
PRISMA: Preferred Reporting Items for Systematic Reviews and Meta–Analyses
qPCR: quantitative Polymerase Chain Reaction
rRNA: Ribosomal RNA
SARI: Severe Acute Respiratory Infections
SISPA: Sequence–Independent Single–Primer Amplification
SMART: Switching Mechanism At the 5’ end of RNA Template
UDG: Uracil–DNA Glycosylase
URTI: Upper Respiratory Tract Infection
VIDISCA: Virus Discovery cDNA–Amplified Fragment Length Polymorphism
WTA: Whole Transcriptome Amplification
