# Supplementary material for: Metagenomic next-generation sequencing for the detection of viruses from respiratory samples: a systematized review on current methods and future directions
Source: Virol J. 2026 Aug 5;23:251. doi: 10.1186/s12985-026-03269-0 (PMC13729278; doi:10.1186/s12985-026-03269-0)
Supplement: Supplementary file 1 [file 12985_2026_3269_MOESM1_ESM.docx]

**Metagenomic next-generation sequencing for the detection of viruses from respiratory samples: A literature review on current methods and future directions**

Pushpa Kumari^a^, Sudheesh N^a*^, Krishna Kumar Ballamoole^b^, Babak Afrough^c^, Anitha Jagadesh^a*^

^a^ Manipal Institute of Virology, Manipal Academy of Higher Education, Manipal, India.

^b^ Department of Infectious Diseases and Microbial Genomics, Nitte University Centre for Science Education and Research, Nitte (Deemed to be University), Mangalore, India

^c^ UK Health Security Agency, United Kingdom

**Correspondence**

1. Anitha Jagadesh, Manipal Institute of Virology, Manipal Academy of Higher Education, Manipal, India. Email: [anitha.j@manipal.edu](mailto:anitha.j@manipal.edu)
2. Sudheesh N, Manipal Institute of Virology, Manipal Academy of Higher Education, Manipal, India. Email: [sudheesh.n@manipal.edu](mailto:sudheesh.n@manipal.edu)

**Co-authors email**

Pushpa Kumari – [pushpa.mivmpl2023@learner.manipal.edu](mailto:pushpa.mivmpl2023@learner.manipal.edu)

Krishna Kumar Ballamoole - [krishnakumarb@nitte.edu.in](mailto:krishnakumarb@nitte.edu.in)

Babak Afrough [-Babak.Afrough@ukhsa.gov.uk](mailto:-Babak.Afrough@ukhsa.gov.uk)

**Methods**

Search formulae of PubMed was listed as below:

#1 (“respiratory virus*” OR “DNA virus” OR “RNA virus”)

#2 (“metagenomic” OR “viral sequenc*” OR “next generation sequenc*” OR “untargeted” OR “unbiased”)

#3 (“clinical sample” OR “respiratory sample”)

#4 #1 AND #2 AND #3

Same search string was used for Scopus and Web of Science

**Results**

**Table S1: Studies on viral metagenomics from respiratory samples (n=66)**

| **Sl No.** | **Author Name (Year)** | **From** | **Subjects** | **Clinical samples** | **Ct values of the positive samples using PCR** | **Number of samples used for mNGS (n)** | **Amplification method** | **Genome coverage** | **Sequencing platform** | **Respiratory viruses detected through mNGS** |
| --- | --- | --- | --- | --- | --- | --- | --- | --- | --- | --- |
| 1. | Christina M. Osborne, et al (2024)(1) | Colorado, US | Critically ill children (1 month to 18 yrs) who required mechanical ventilation | NPS, TA | Not mentioned | 295 | dUTP-based strand-specific amplification -  Kit based (NEBNext Ultra II Directional RNA Library prep kit for Illumina) | Not mentioned | Illumina, HiSeq 4000 | 1. Respiratory syncytial virus 2. Rhinovirus/enterovirus 3. Adenovirus 4. Coronavirus 5. Human metapneumovirus 6. Influenza A/B virus 7. Parainfluenza 1, 2, 3, 4 |
| 2. | de Campos GM, et al (2024)(2) | Brazil | ARI (mostly LRTI) | NPS | Not mentioned | 66 | MDA -  Kit based (Quantitect WTA) | Not mentioned | Illumina NextSeq 2000 | 1. Rhinoviruses (A & B) 2. Enterovirus D68 3. Human parainfluenza 3, 4. Herpesviruses 5. Human parvovirus B19 6. Human vientovirus FB 7. Anelloviruses |
| 3. | Nurlan Sandybayev, et al  (2024)(3) | Kazakhstan | Adults with ARI (URTI) | NPS | Not mentioned | 53 | Random priming-based whole transcriptome amplification -Kit based  (SeqPlex RNA Amplification Kit) | Not mentioned | Ion Torrent S5 System | 1. Rhinovirus-A 2. Betaherpesvirus 3. Epstein-Barr virus |
| 4. | Jessica Karielle Tan, et al (2024)(4) | USA | Not reported | Upper respiratory swab, BAL | Not mentioned | 191 | dUTP-based strand-specific amplification -Kit based (NEBNext Ultra II Directional RNA Library prep kit for Illumina) | Not mentioned | Illumina MiniSeq or Illumina NextSeq 550 | 1. Respiratory syncytial virus A 2. Influenza C 3. Human rubulavirus 4 4. Human coronavirus NL63 5. Human coronavirus 229E 6. Human bocavirus 1 7. Parechovirus A1 8. Rhinovirus A, B, C 9. Enterovirus D 10. Coxsackievirus A |
| 5. | Nick P. G. Gauthier, et al (2024)(5) | Canada | Not reported | NPS | <30 | 359 | Rapid SMART (9N) | Not mentioned | Nanopore, GridION | 1. Human metapneumovirus 2. Human coronavirus HKU1 3. Human coronavirus OC43 4. Human parvovirus B19 5. Human mastadenovirus B 6. Human papillomavirus (type 129) 7. Parainfluenza virus 2,3 8. Rhinovirus C |
| 6. | Makoto Yamaguchi, et al  (2023)(6) | Japan | Children with SARI | BALF | Not mentioned | 31 | MDA –  Kit based (REPLI-g WTA Single Cell kit) | 44.7 to 100% | Nanopore (PromethION) | 1. Human metapneumovirus 2. Respiratory syncytial virus -A 3. Rhinovirus 4. Enterovirus D-68 |
| 7. | de Campos GM, et al (2023)(7) | Brazil | Children – ARI (mild to moderate) | NPS | Not mentioned | 96 | MDA –  Kit based (Quantitect WTA) | Not mentioned | Illumina NextSeq 2000 | 1. Respiratory syncytial virus 2. Enterovirus 3. Mumps 4. Orthorubeolavirus 5. Human metapneumovirus 6. Influenza A 7. Human herpes viruses 8. Anelloviruses |
| 8. | Ingra M. Claro, et al (2023)(8) | Brazil | Not reported | NPS | 21.8 to 33.3 | 10 | Rapid SMART (9N) | 95.90% to 99.92% | Nanopore | 1. SARS-CoV-2 |
| 9. | Rachel L. Tulloch, et al (2023) (9) | Australia | Children with SARI | NPS, NPA | 17.18 to 33.92 | 34 | SISPA-6N | >90% for RSV and >99.9% for SARS-CoV-2 | Illumina, MiSeq | 1. SARS-CoV-2 2. Respiratory syncytial virus A & B 3. Flu A – pdmH1N1 4. Flu C 5. Rhinovirus 6. GB virus C 7. Cytomegalovirus 8. HHV-7 |
| 10. | Anielly S. da Silva, et al (2023)(10) | Brazil | Children <12 years, Acute respiratory disease | NPS | Not mentioned | 96 | MDA –  Kit based (Quantitect WTA) | Not mentioned | Illumina NextSeq 2000 | 1. Respiratory syncytial virus 2. Human metapneumovirus 3. Rhinovirus A, B, C, NAT00 4. Enteroviruses A, B, D, F, H, K, L 5. Human herpesvirus types 1, 4, 5, 6, and 7 6. Human papillomavirus 5 7. Torque tenovirus 8. Polyomavirus |
| 11. | Ayodeji E. Ogunbayo, et al (2023)(11) | South Africa | Children with SARI | NPS | 11.45 to 35.01 | 84 | MDA –  Kit based QIAseq FX Single Cell RNA Library Kit | 0.13 to 98.86% | Illumina, MiSeq | 1. Respiratory syncytial virus 2. Human rhinovirus A, C 3. SARS-CoV-2 |
| 12. | Ayodeji Emmanuel Ogunbayo, et al (2023)(12) | South Africa | Not reported | NPS | 18.6 to 34.8 | 1 | MDA –  Kit based QIAseq FX Single Cell RNA Library Kit | 6 to 100% | Illumina, MiSeq | 1. Human rhinovirus 2. Respiratory syncytial virus 3. SARS-CoV-2 4. Human metapneumovirus 5. Influenza virus 6. Human parainfluenza virus-3 |
| 13. | Stacey Cerón, et al  (2023)(13) | USA | Solid organ transplant patients and deceased ICU patients | NPS or BAL | 11.45-35.06 | 40 | dUTP-based strand-specific amplification - Kit based (NEBNext Ultra II Directional RNA Library prep kit for Illumina) | 34 to 100% | Illumina, Miseq | 1. SARS-CoV-2 |
| 14. | Antonin Bal, et al  (2022)(14) | France | Children with ARI | NPS | Not mentioned | 27 | Whole transcriptome amplification -Kit based (Complete Whole Transcriptome Amplification Kit) WTA2 | Not mentioned | Illumina | 1. Respiratory syncytial virus 2. Influenza 3. Torque tenovirus |
| 15. | Anna S. Fomsgaard, et al  (2022)(15) | Denmark, Europe | Not reported | OPS | 18 to 36 and one negative sample (>38 Ct value were included | 15 | MDA –  Repli-g WTA Single Cell kit Qiagen | 36.8-100.0% | Nanopore | 1. SARS-CoV-2 |
| 16. | Yoong Min Chong, et al (2022)(16) | Malaysia | Adults with SARI (LRTI) | NPS, OPS, sputum, or BAL | Not mentioned | 105 | SISPA (8N) | 9.9 to 99.5% | Illumina NextSeq 500 | 1. Rhinovirus 2. Enterovirus 3. Influenza virus |
| 17. | My V.T. Phan, et al  (2022)(17) | UK | Severe pneumonia | NPS/OPS | Not mentioned | 190 | SISPA – 6N | Not mentioned | Illumina, HiSeq 2500 | 1. Human rhinovirus A, B, C 2. Human metapneumovirus 3. Respiratory syncytial virus B 4. Human parainfluenza virus-1 5. Human Herpes Virus-5 6. Herpes simplex virus-1 7. Echovirus E1 8. Parechovirus 9. Enterovirus, species A, B, C, D  10. Human poliovirus 2 11. Human Bocavirus  12. Parvovirus B19 13. Rubella virus |
| 18. | Pavel Iša, et al  (2022)(18) | Mexico | Not reported | NPS, OPS and tracheal aspirates | Not mentioned | 120 | SISPA (9N) | 0.1 to 100% | Illumina, NextSeq 500 | 1. SARS-CoV-2 2. Enterovirus B, C 3. Human bocavirus 2. Human mastadenovirus C, F 3. Human Coronavirus HKU1 4. Rhinovirus A, B, C 5. Influenza A, B  6. Metapenumovirus 7. Anelloviridae species 8. Measles 9. Herpesviruses |
| 19. | Qiong Guo, et al  (2022)(19) | China | RTI in children under 6 years | NPS | Not mentioned | 185 | MDA **-**Kit-based (Quantitect WTA)  Kit based (cDNA synthesis)using oligo(dT)20 and random hexamers and subsequent amplification by kit | Not mentioned | Illumina, Hiseq 2000 | 1. Human respiratory syncytial virus 2. Human rhinovirus 3. Human polyomavirus 4. Influenza virus 5. Human adenovirus 6. Human parainfluenza virus 7. Human bocavirus 8. Human coronavirus |
| 20. | Qingqing Mao, et al (2022)(20) | China | ARI in children under 5 years (URTI) | Pharyngeal swab | Not mentioned | 411 | SISPA (6N) | 82 to 100% | Illumina, HiSeq | 1. Human bocavirus 2. Human Enterovirus A, B 3. Anelloviridae 4. Papillomaviridae 5. Parvoviridae 6. Paramyxoviridae 7. Picobirnaviridae |
| 21. | Ying Liu, et al (2021)(21) | China | ARI in children | NPS/OPS | Not mentioned | 30 | Unclear | Not mentioned | Illumina, MiSeq | 1. Human parainfluenza virus 1 2. Anellovirus 3. Bocavirus 4. Coxsackievirus 5. Human parechovirus 6. Alphaflexivirus |
| 22. | Xiaofang Jia, et al  (2021)(22) | China | Not reported | Throat swab, sputum | 19.02 to 33.1 | 11 | SMART (6N) | 2.03 to 98.75% | Illumina, NovaSeq 6000 | 1. Flu A (H1N1) 2. Flu A (H3N2) 3. Human Rhinovirus 4. Human Adenovirus 5. Human coronavirus 6. Human adenovirus B1 |
| 23. | Yifei Xu, et al (2021)(23) | UK | Not reported | Throat swabs, nasal swabs, and NPA | 15.4 to 39.0 | 180 | SISPA (9N) | 2.7 to 100% | Nanopore | 1. Influenza A 2. Human metapneumovirus 3. Respiratory syncytial virus 4. Parainfluenza 5. Coronavirus 6. Enterovirus |
| 24. | Jacqueline Wahura Waweru, et al (2021)(24) | Kenya | Severe pneumonia in children below 5 years | NPS | <24 | 32 | SISPA (6N) | Not mentioned | Nanopore | 1. Respiratory syncytial virus |
| 25. | Yoong Min Chong, et al (2021)(25) | Malaysia | Adult patients (≥ 18 yrs) with suspected COVID-19 (URTI & LRTI) | Single combined oro-nasopharyngeal swab | Not mentioned | 45 | SISPA (8N) | Enterovirus D68 - 25.7%  Saffold virus - 87.1%  Human papillomavirus - 66.6% | Illumina, NextSeq 500 | 1. Scaffold virus 2. Enterovirus D68 3. Human Papillomavirus type 4 |
| 26. | Nick P. G. Gauthier, et al (2021)(26) | Canada | Not reported | NPS | 14.7–38.7 | 43 | SISPA (9N) | At 1X coverage - 3.63 to 100% | Nanopore | 1. SARS-CoV-2 |
| 27. | Marina Sabatier, et al (2020)(27) | France | Not reported | BAL and NPA | 16.6 and 19.3 | 2 | WTA –  Kit based (Complete Whole Transcriptome Amplification Kit) WTA2 | Not mentioned | Illumina, NextSeq 500 | 1. Respiratory syncytial virus 2. Herpes simplex virus |
| 28. | Nguyen Thi Kha Tu, et al (2020)(28) | Finland, Europe | Acute respiratory infection (URTI among adults and children) | Throat swabs | 29.3 to 40 | 91 | SISPA (6N) | 0.8 to 100% | Illumina, MiSeq | 1. Enterovirus-D68 2. Human rhinovirus B, C 3. Influenza A virus 4. Respiratory syncytial virus 5. Human coronavirus- OC43 6. Rotavirus 7. Anellovirus |
| 29. | Lu Lu, et al (2020)(29) | UK | Patients (Adults & children) with Acute RTI | Nasal and throat swabs | Not mentioned | 300 | SISPA (6N) | Not mentioned | Illumina, MiSeq | 1. Respiratory syncytial virus 2. Human rhinovirus 3. Influenza A virus 4. Human bocavirus 5. Human parainfluenza 1, 2, 3, 4 |
| 30. | Vikas Peddu, et al  (2020)(30) | USA | Not reported | NPS | 20.7 to 29.5 | 8 | SISPA (8N) | 97.6% to 99.8% | Illumina, MiSeq | 1. SARS-CoV-2 2. Human parainfluenza 3 3. Rhinovirus |
| 31. | Ci-Xiu Li, et al  (2020)(31) | China | Children with ARI (URTI & LRTI) | Throat swabs | Not mentioned | 48 | SMART –  Kit based (TaKaRa) SMARTer Stranded Total RNA-Seq kit v.2 and  SPIA –  Kit based Trio RNA-Seq kit (NuGEN Technologies, USA)  (No samples used both the methods) | Not mentioned | Illumina, HiSeq | 1. Human rhinovirus A, C 2. Human coronavirus-HKU1 3. Influenza A H1N1, B 4. Human metapneumovirus 5. Respiratory syncytial virus 6. Measles  7. Parainfluenza-3 8. cytomegalovirus  9. Epstein barr virus 10. Epstein barr virus 11. Human herpes virus 1 12. Human papillomavirus |
| 32. | Fan Wu, et al (2020)(32) | China | Patients (Adults) with respiratory illness | BALF | Not mentioned | 1 | SMART -  Kit based (TaKaRa) SMARTer Stranded Total RNA-Seq kit v.2 | 99.9% | Illumina, MiniSeq | 1. SARS-CoV-2 |
| 33. | Bo Liu, et al (2020)(33) | China | Hospitalized patients with COPD (Acute LRTI) | Pharyngeal swab | Not mentioned | 3 | SISPA (8N) | 97.8–99.9% for coronavirus | Illumina, MiniSeq | 1. Coronavirus OC43 2. Anelloviruses |
| 34. | Yifei Xu, et al (2020)(34) | UK | Haematology and immunocompromised patients | Throat swab | Not mentioned | 25 | SISPA (9N) | 10.4 to 100% | Nanopore | 1. Human metapneumovirus |
| 35. | Heba H. Mostafa, et al (2020)(35) | USA | COVID patients (URTI & LRTI) | NPS | 14.2 to 36.6 | 50 | Kit based (Oxford Nanopore Technologies) Poly A and random amplification | 0.5 to 100% | Nanopore | 1. SARS-CoV-2 2. Human metapneumovirus 3. human alphaherpesvirus 1 |
| 36. | Caroline Charre, et al (2020)(36) | France | Not reported | NPS | 10.7 to 33.9 | 24 | WTA –  Kit based (Complete Whole Transcriptome Amplification Kit) WTA2 | 0.7 to 100% | Illumina NextSeq 550 | 1. SARS-CoV-2 |
| 37. | Ellen C. Carbo, et al (2020)(37) | Netherlands | Not reported | NPA | 20 and 30 | 2 | dUTP-based strand-specific amplification –  Kit based (NEBNext Ultra II Directional RNA Library prep kit for Illumina) | 74.2% and 98.4% | Illumina, NovaSeq 6000 | 1. SARS-CoV-2 2. SARS-CoV 3. MERS |
| 38. | Sander van Boheemen, et al  (2019)(38) | Netherlands | Paediatric patients | NPW, or sputa, or BAL, or Bronchial washings, throat swab | 23.1 to 37.7 | 25 | dUTP-based strand-specific amplification - Kit based (NEBNext1 Ultra™ Directional RNA Library Prep Kit for Illumina) | 99% for influenza C | Illumina HiSeq 4000 and NextSeq 500 | 1. Human rhinovirus 2. Human adenovirus 3. Human coronavirus 4. Influenza A, B, C virus 4. Human metapneumovirus 5. Human bocavirus 6. KI polyomavirus 7. cytomegalovirus 8. Enterovirus |
| 39. | Unai Pérez-Sautu, et al (2019)(39) | Spain | Paediatric patients with respiratory infections (URTI/LRTI) | NPA | Not mentioned | 127 | SISPA (8N) | Not mentioned | Illumina, MiSeq or Illumina, NextSeq 500 | 1. Human orthopneumovirus A, B 2. Human metapneumovirus 2. Enterovirus B, D-68 3. Rhinovirus A, B, C 4. Human coronavirus-HKU1, NL63, OC43 5. Influenza B virus  6. Human parechovirus 1 7. Human rubulavirus 4 8. Human papillomavirus 9. Anelloviruses 10. Human polyomavirus 4  11. Rotavirus A 12. Human betaherpesvirus 5 13. Respiratory syncytial virus 14. Enterovirus 15. Human parainfluenza virus |
| 40. | Kuiama Lewandowski, et al  (2019)(40) | UK | Not reported | NPS/OPS | 12 to 40 | 159 | SISPA (9N) | 10 to >90% | Nanopore | 1. Influenza A virus 2. Human coronavirus 3. Human metapneumovirus |
| 41. | Anneloes L. van Rijn, et al (2019)(41) | Netherlands | Patients with COPD (LRTI) | NPS | 19 to 38 | 88 | dUTP-based strand-specific amplification - Kit based (NEBNext1 Ultra™ Directional  RNA Library Prep Kit for Illumina) random primers" | 3 to 100% | Illumina NextSeq 500 | 1. Influenza A virus 2. Human adenovirus 2. Human coronavirus- NL63, OC43 3. Human parainfluenza virus-3 , 4 4. Rhinovirus |
| 42. | Talia Kustin, et al (2019)(42) | Israel | ILI patients | combined nose throat swab samples | Ct value >35 | 31 | SMART –  Kit based (TaKaRa) SMARTer Stranded Total RNA-Seq kit v.2 | Not mentioned | Illumina, MiSeq | 1. Human adenovirus C 2. Respiratory syncytial virus 3. Influenza B virus 4. Human parainfluenza 1 5. Anellovirus 6. Human herpes virus 4 7. Human papillomavirus |
| 43. | A. Bal, et al (2018)(43) | France | Patients with ARI | NPS, or NPA, or sputum | 15 to 37 | 37 | WTA –  Kit based (Complete Whole Transcriptome Amplification Kit) WTA2 | 0.6 to 100% | Illumina, NextSeq 500 | 1. Human rhinovirus-A 2. Respiratory synctial virus A, B 3. Human metapneumovirus A 4. Human coronavirus-NL63, 229E,  5. Measles virus 6. Influenza A (H3N2), B virus 7. Human adenovirus 8. Human parainfluenza virus-3 9. Human bocavirus-1 10. Human herpes virus 6B 11. Cytomegalovirus 12. Epstein barr virus |
| 44. | Nada Madi, et al  (2018)(44) | Kuwait | Patients with URTI and LTRI | NPA, NPS, Sputum, throat swabs, BAL, TA, Nasal swabs | 10.36 to 33.91 | 86 | MDA –  Kit based (Quantitect WTA) | 1.01 to 99.23% | Illumina, MiSeq | 1. Influenza A (H9N2), (H2N2), (H1N1), (H5N1), C 2. Rhinovirus 3. Respiratory synctial virus 4. Human metapneumovirus 5. Coronavirus-HKU14 6. Anellovirus 7. Rotavirus A, E, C 8. Human bocavirus 9. Hantavirus |
| 45. | Jose A. Romero-Espinoza, et al  (2018)(45) | Mexico | Children (<15 yrs) with ARI (LRTI) | NPS | Not mentioned | 127 | SISPA (6N) | 7.8% to 97% | Illumina, MiSeq | 1. Rhinovirus C 2. Bocavirus 2. Influenza A virus 2. Respiratory synctial virus 3. Parainfluenza virus 4. Adenovirus 6. Parvovirus B19 7. Anellovirus 8. Enterovirus D-68 9. Cytomegalovirus 10. Coxsackie virus |
| 46. | Colin Sharp, et al  (2018)(46) | UK | Not reported | NPA, BAL, ETA | Not mentioned | 10 | SISPA (8N) | Not mentioned | Illumina, HiSeq 4000 | 1. Human rhinovirus 2. Respiratory syncytial virus 3. Human metapneumovirus 4. Human herpes virus -6 |
| 47. | Stephanie Goya, et al (2018)(47) | Argentina | Children (< 2yrs) with acute LRTI | NPA | Not mentioned | 20 | SISPA (8N) | 51.72% - 100% | Illumina, Miseq  Illumina, NextSeq 500 | 1. Human rhinovirus 2. Human metapneumovirus 3. Human parainfluenza virus1, 2, 3 4 Human rhinovirus |
| 48. | Dan Zhang, et al  (2018)(48) | China | Not reported | NPA | 15.8 to 38.8 | 2 | SISPA (8N) | 0.23 to 100% | Illumina, HiSeq2500 | 1. Human adenovirus 2. Influenza A virus 3. Human parainfluenza 3 4. Human coronavirus 5. Human parainfluenza virus |
| 49. | Xiaohui Zou, et al  (2017)(49) | China | Unexplained pneumonia | OPS | Not mentioned | 33 | SPIA –  Kit based (Ovation RNASeq) | 2.58 to 100% | Ion PGM™ and Illumina Miseq (for scant cDNA) | 1. Influenza B virus 2. Rhinovirus A, C 3. Adenovirus B 4. Human parainfluenzavirus-3 5. Human coronavirus-HKU1, OC43, 229E 6. Respiratory syncytial virus 7. Human Papillomavirus 8. Coxsackie virus 9. Measles virus 10. Anellovirus 11. Human endogenous retrovirus |
| 50. | Alexander L, et al  (2017)(50) | USA | Patients hospitalized on the general medical unit who met criteria for hospital-acquired HPIV3 | NPS | Not mentioned | 13 | SISPA (8N) | Not mentioned | Illumina, Miseq | 1. Human parainfluenza 3 |
| 51. | Yu-Nong Gong, et al (2017)(51) | Taiwan | Children with RTI | Throat swabs, NPS, sputum | Not mentioned | 40 | SPIA -  Kit based (Ovation RNASeq) | 3.5 to 100% | Illumina, MiSeq | 1. Human rhinovirus 2. Human coronavirus NL63 3. Human parainfluenza virus - 4a 4. Human parechovirus 5. Influenza A virus 6. Respiratory syncytial virus 7. Human metapneumovirus |
| 52. | Dagmara W. Lewandowska, et al (2017)(52) | Switzerland | Adults >18 years (lung transplant receipient) | Throat swabs/BAL | 17.12 to 44.4 | 113 | SISPA (8N) | Not mentioned | Illumina MiSeq | 1. Rhinovirus A, B 2. Coronavirus 3. Anelloviruses 4. KI polyomavirus, 5. Human papillomavirus 6. Human herpesvirus 6A and 6B |
| 53. | Lunbiao Cui, et al (2017)(53) | China | Adult with AFI | Throat swab | Not available | 1 | SISPA (9N) and MDA  (No samples used both the methods) | Not mentioned | Illumina MiSeq | 1. human respiratory-associated PSCV-5-like virus (HRAPLV) |
| 54. | Y. Wang, et al (2016)(54) | China | Children (<6 yrs) with SARI and without SARI | NPS | Not available | 150 | SISPA (6N) | 51.5% to 100% | Illumina Hiseq 2500 | 1. Human respiratory syncytial virus 2. Human parainfluenza virus-1, 3 3. Human metapneumovirus 4. Human coronavirus - OC43, HKU1, 229E 5. Influenza A, B, C virus 6. Human rhinovirus 7. Human bocavirus 8. Human adenovirus 9. Anelloviruses 10. Adenovirus |
| 55. | Erin H. Graf, et al  (2016)(55) | USA | Children less than 5 yrs | NPS | Not available | 109 | Poly A capture-based random amplification - Kit based (oligo dT and random hexamers - trueSeq RNA sample prep kit) | Not mentioned | Illumina, HiSeq 2500 | 1. Human rhinovirus 2. Human metapneumovirus 3. Influenza A, B virus 4. Parainfluenza virus 1, 2, 3, 4 5. Human respiratory syncytial virus 6. Human coronavirus 7. Human bocavirus 8. Enterovirus  9. Measles virus 10. Cytomegalovirus |
| 56. | Fiona Thorburn, et al  (2015)(56) | UK | Adults with URTI | NPS | 17.21 to 36.07 | 89 | 2.6 to 100% | 2.6 to 100% | Illumina, MiSeq | 1. Enterovirus D-68 2. Human coronavirus 229E, NL63, OC43 3. Human metapneumovirus B 4. Human rhinovirus  5. Influenza A (H3N2) 6. Parainfluenza virus – 3 7. Respiratory syncytial virus A, B |
| 57. | Jan Zoll, et al (2015)(57) | Netherlands | Children with RTI (moderate to severe) | NPA, Sputum | 25 to >40 | 10 | MDA –  Kit-based (WGA & WTA) Repli-G Cell WGA and WTA | 38% to 99% | IonTorrent PGM system | 1. Human Rhinovirus C 2. Entero and rhinovirus 3. Human metapneumovirus 4. Anellovirus 5. Respiratory syncytial virus |
| 58. | F. M. Moesker, et al (2015)(58) | Germany | SARI – Paediatric (<18 years) | Nasal washings, Sputum, throat swabs, broncheoalveolar lavages | 11.6–16.3 | 24 | SISPA (6N) | Not mentioned | Roche 454 sequencing | 1. Bocavirus  2. Respiratory syncytial virus  3. Rhinovirus |
| 59. | Nicole Fischer, et al (2015)(59) | Germany | ARI | BAL, sputum samples and a swab, Tracheal secretions | 23 to 40 | 29 | Similar to SMART –  Kit based (ScriptSeq V2) Random Hexamer with Tagging Sequence | Not mentioned | Illumina, Miseq or HiSeq 2500 | 1. Influenza A virus 2. Human respiratory syncytial virus 3. Rhinovirus A 4. Human herpes virus 1, 7 5. Parainfluenza 3 6. Anellovirus 7. human Merkel cell polyomavirus |
| 60. | Weihua Huang, et al (2015)(60) | USA | Severe respiratory illness among children | NPS | 18.11 to 39.76 | 85 | No amplification method used | Not mentioned | Illumina, Miseq | 1. Enterovirus-D68 |
| 61. | Jacque C, et al (2015)(61) | Pennsylvania | Lung transplant recipients, HIV infected individual and healthy individuals | BAL, Oropharyngeal wash | Not available | 21 | MDA –  Kit based  (GenomiPhi V2 Amplification Kit) | Not mentioned | Illumina MiSeq | 1. Anelloviruses 2. Epstein barr virus, Human herpes virus 7, and human papillomavirus were detected in lower level |
| 62. | Blanca Taboada, et al (2014)(62) | Mexico | Children with LTRI/UTRI | Nasal washings and NPS | Not available | 71 | SISPA (9N) | LRTI - 0.40 to 99.81%  URTI - 0.13% to 97.88% | Illumina | 1. Respiratory syncytial virus 2. Human Coronavirus-OC43 3. Rhinovirus A, B, C 4. Human Bocavirus 5. Anelloviruses 6. Rotavirus, Human Papillomavirus, herpesvirus, enterovirus A71, astrovirus 7. Scaffold virus |
| 63. | David E. Alquezar-Planas, et al (2013)(63) | Denmark | Children with URTI/LTRI (0-5 yrs) | NPA and tracheal secretions | Not available | 92 | SISPA (8N) | 98% (for divergent HPIV-4) | 454 sequencing GS Flex, PacBio | 1. Human parainfluenza 4 2. Human metapneumovirus 3. Influenza A virus 4. Human parainfluenza 3 5. Adenovirus 6. Human rhinovirus A, C 7. Human coronavirus-OC43 |
| 64. | Fredrik Lysholm, et al (2012)(64) | Sweden | Patients (mixed) with Severe LTRI | NPA | Not available | 210 | SISPA (6N) | Not mentioned | 454 sequencing  GS20  GS Flex | 1. Human respiratory syncytial virus 2. Influenza A, B, C virus 3. Human rhinovirus A, C 4. Human parainfluenza 3 5. Human coronavirus - HKU1, OC43 6. Human metapneumovirus 7. Human Bocavirus 8. Anelloviruses 9. Measles virus 10. Circovirus 11. KI polyomavirus 12. Human adenovirus C |
| 65. | Jian Yang, et al  (2011)(65) | China | Children ( 2 months to 10 years) with acute LTRI (admitted) | NPA | Not available | 16 | SMART | 21.84% to 98.53% | Illumina Solexa GA II | 1. Human adenovirus  2. Human respiratory syncytial virus 3. Human rhinovirus 4. Influenza virus A, B 5. Parainfluenza 3 6. Human bocavirus 7. Human enterovirus B 8. Human herpes virus 5 |
| 66. | Michel de Vries, et al (2011)(66) | Netherlands | Acute cough in adults | NPS | 1.4 E3–7.7 E6 viral copies/mL | 18 | VIDISCA (Virus discovery cDNA-AFLP) | 0.3 to 79% | Roche 454 GS  FLX Titanium sequencing | 1. Respiratory syncytial virus 2. Influenza A, B 3. Human Coronavirus-NL63 4. Adenovirus 5. Human Coronavirus-OC43 |

(LRTI – Lower Respiratory Tract Infection, URTI – Upper Respiratory Tract Infection, ARI – Acute Respiratory Infection, RTI – Respiratory Tract Infection, NPA – Nasopharyngeal aspirates, NPS – Nasopharyngeal swabs, SMART – Switching Mechanism at the 5’ end of RNA Template, SISPA – Sequence independent single-primer amplification)

**RESULTS:**

**Characteristics of the studies:**

1. **Year of publication:**

a

Year-wise studies on untargeted sequencing of respiratory samples

| Year of publication | Percentage of publications |
| --- | --- |
| 2011-2014 | 7.5 |
| 2015 | 9 |
| 2016 | 3 |
| 2017 | 7.5 |
| 2018 | 9 |
| 2019 | 7.5 |
| 2020 | 16.6 |
| 2021 | 9 |
| 2022 | 10.6 |
| 2023 | 12 |
| 2024 | 7.5 |

**Figure 1: A bar graph representing the percentage of publications in the particular year**

**REFERENCES:**

1.	Osborne CM, Langelier C, Kamm J, Williamson K, Ambroggio L, Reeder RW, et al. Viral Detection by Reverse Transcriptase Polymerase Chain Reaction in Upper Respiratory Tract and Metagenomic RNA Sequencing in Lower Respiratory Tract in Critically Ill Children With Suspected Lower Respiratory Tract Infection. Pediatric Critical Care Medicine. 2024 Jan;25(1):e1–11.

2.	De Campos GM, Santos HDO, Lima ARJ, Leite AB, Ribeiro G, Todão Bernardino JDS, et al. Unveiling viral pathogens in acute respiratory disease: Insights from viral metagenomics in patients from the State of Alagoas, Brazil. Schibler M, editor. PLoS Negl Trop Dis. 2024 Sep 23;18(9):e0012536.

3.	Sandybayev N, Beloussov V, Strochkov V, Solomadin M, Granica J, Yegorov S. Metagenomic profiling of nasopharyngeal samples from adults with acute respiratory infection. R Soc Open Sci. 2024 Jul;11(7):240108.

4.	Tan JK, Servellita V, Stryke D, Kelly E, Streithorst J, Sumimoto N, et al. Laboratory validation of a clinical metagenomic next-generation sequencing assay for respiratory virus detection and discovery. Nat Commun. 2024 Nov 12;15(1):9016.

5.	Gauthier NPG, Chan W, Locher K, Smailus D, Coope R, Charles M, et al. Validation of an Automated, End-to-End Metagenomic Sequencing Assay for Agnostic Detection of Respiratory Viruses. The Journal of Infectious Diseases. 2024 May 2;jiae226.

6.	Yamaguchi M, Horiba K, Haruta K, Takeuchi S, Suzuki T, Torii Y, et al. Utility of nanopore sequencing for detecting pathogens in bronchoalveolar lavage fluid from pediatric patients with respiratory failure. Journal of Clinical Virology Plus. 2023 Jun;3(2):100154.

7.	De Campos GM, De La-Roque DGL, Lima ARJ, Zucherato VS, De Carvalho E, De Lima LPO, et al. Exploring Viral Metagenomics in Pediatric Patients with Acute Respiratory Infections: Unveiling Pathogens beyond SARS-CoV-2. Microorganisms. 2023 Nov 10;11(11):2744.

8.	Claro IM, Ramundo MS, Coletti TM, da Silva CAM, Valenca IN, Candido DS, et al. Rapid viral metagenomics using SMART-9N amplification and nanopore sequencing. Wellcome Open Res. 2021;6:241.

9.	Tulloch RL, Kim K, Sikazwe C, Michie A, Burrell R, Holmes EC, et al. RAPIDprep: A Simple, Fast Protocol for RNA Metagenomic Sequencing of Clinical Samples. Viruses. 2023 Apr 19;15(4):1006.

10.	Da Silva AS, De Campos GM, Giovanetti M, Zucherato VS, Lima ARJ, Santos EV, et al. Viral metagenomics unveils MW (Malawi) polyomavirus infection in Brazilian pediatric patients with acute respiratory disease. Journal of Medical Virology. 2023 Apr;95(4):e28688.

11.	Ogunbayo AE, Mogotsi MT, Sondlane H, Sabiu S, Nyaga MM. Metagenomics characterization of respiratory viral RNA pathogens in children under five years with severe acute respiratory infection in the Free State, South Africa. Journal of Medical Virology. 2023 May;95(5):e28753.

12.	Ogunbayo AE, Sabiu S, Nyaga MM. Evaluation of extraction and enrichment methods for recovery of respiratory RNA viruses in a metagenomics approach. Journal of Virological Methods. 2023 Apr;314:114677.

13.	Cerón S, Clemons NC, Von Bredow B, Yang S. Application of CRISPR-Based Human and Bacterial Ribosomal RNA Depletion for SARS-CoV-2 Shotgun Metagenomic Sequencing. American Journal of Clinical Pathology. 2023 Feb 1;159(2):111–5.

14.	Bal A, Destras G, Sabatier M, Pichon M, Regue H, Oriol G, et al. Metagenomic Analysis Reveals High Abundance of Torque Teno Mini Virus in the Respiratory Tract of Children with Acute Respiratory Illness. Viruses. 2022 May 3;14(5):955.

15.	Fomsgaard AS, Rasmussen M, Spiess K, Fomsgaard A, Belsham GJ, Fonager J. Improvements in metagenomic virus detection by simple pretreatment methods. Journal of Clinical Virology Plus. 2022 Nov;2(4):100120.

16.	Chong YM, Chan YF, Jamaluddin MFH, Hasan MS, Pang YK, Ponnampalavanar S, et al. Rhinovirus/enterovirus was the most common respiratory virus detected in adults with severe acute respiratory infections pre-COVID-19 in Kuala Lumpur, Malaysia. PLOS ONE.

17.	Phan MVT, Agoti CN, Munywoki PK, Otieno GP, Ngama M, Kellam P, et al. Identification of missed viruses by metagenomic sequencing of clinical respiratory samples from Kenya. Sci Rep. 2022 Jan 7;12(1):202.

18.	Iša P, Taboada B, García-López R, Boukadida C, Ramírez-González JE, Vázquez-Pérez JA, et al. Metagenomic analysis reveals differences in the co-occurrence and abundance of viral species in SARS-CoV-2 patients with different severity of disease. BMC Infect Dis. 2022 Oct 19;22(1):792.

19.	Guo Q, Li L, Wang C, Huang Y, Ma F, Cong S, et al. Comprehensive virome analysis of the viral spectrum in paediatric patients diagnosed with Mycoplasma pneumoniae pneumonia. Virol J. 2022 Nov 9;19(1):181.

20.	Mao Q, Sun G, Qian Y, Qian Y, Li W, Wang X, et al. Viral metagenomics of pharyngeal secretions from children with acute respiratory diseases with unknown etiology revealed diverse viruses. Virus Research. 2022 Nov;321:198912.

21.	Liu Y, Wang H, Yang J, Zeng J, Sun GM. Virome of respiratory secretion from children with unknown etiological acute respiratory disease revealed recombinant human parechovirus and other significant viruses. Virol J. 2021 Dec;18(1):122.

22.	Jia X, Hu L, Wu M, Ling Y, Wang W, Lu H, et al. A streamlined clinical metagenomic sequencing protocol for rapid pathogen identification. Sci Rep. 2021 Feb 23;11(1):4405.

23.	Xu Y, Lewandowski K, Downs LO, Kavanagh J, Hender T, Lumley S, et al. Nanopore metagenomic sequencing of influenza virus directly from respiratory samples: diagnosis, drug resistance and nosocomial transmission, United Kingdom, 2018/19 influenza season. Eurosurveillance [Internet]. 2021 Jul 8 [cited 2024 Nov 26];26(27). Available from: https://www.eurosurveillance.org/content/10.2807/1560-7917.ES.2021.26.27.2000004

24.	Waweru JW, De Laurent Z, Kamau E, Mohammed KS, Gicheru E, Mutunga M, et al. Enrichment approach for unbiased sequencing of respiratory syncytial virus directly from clinical samples. Wellcome Open Res. 2021 May 7;6:99.

25.	Chong YM, Chan YF, Jamaluddin MFH, Hasan MS, Pang YK, Ponnampalavanar S, et al. Detection of respiratory viruses in adults with suspected COVID-19 in Kuala Lumpur, Malaysia. Journal of Clinical Virology. 2021 Dec;145:105000.

26.	Gauthier NPG, Nelson C, Bonsall MB, Locher K, Charles M, MacDonald C, et al. Nanopore metagenomic sequencing for detection and characterization of SARS-CoV-2 in clinical samples. PLOS ONE. 2021 Nov 18;16(11):e0259712.

27.	Sabatier M, Bal A, Destras G, Regue H, Quéromès G, Cheynet V, et al. Comparison of Nucleic Acid Extraction Methods for a Viral Metagenomics Analysis of Respiratory Viruses. Microorganisms. 2020 Oct 6;8(10):1539.

28.	Thi Kha Tu N, Thi Thu Hong N, Thi Han Ny N, My Phuc T, Thi Thanh Tam P, Doorn HRV, et al. The Virome of Acute Respiratory Diseases in Individuals at Risk of Zoonotic Infections. Viruses. 2020 Aug 29;12(9):960.

29.	Lu L, Robertson G, Ashworth J, Pham Hong A, Shi T, Ivens A, et al. Epidemiology and Phylogenetic Analysis of Viral Respiratory Infections in Vietnam. Front Microbiol. 2020 May 15;11:833.

30.	Peddu V, Shean RC, Xie H, Shrestha L, Perchetti GA, Minot SS, et al. Metagenomic Analysis Reveals Clinical SARS-CoV-2 Infection and Bacterial or Viral Superinfection and Colonization. Clinical Chemistry. 2020 Jul 1;66(7):966–72.

31.	Li CX, Li W, Zhou J, Zhang B, Feng Y, Xu CP, et al. High resolution metagenomic characterization of complex infectomes in paediatric acute respiratory infection. Sci Rep. 2020 Mar 3;10(1):3963.

32.	Wu F, Zhao S, Yu B, Chen YM, Wang W, Song ZG, et al. A new coronavirus associated with human respiratory disease in China. Nature. 2020 Mar 12;579(7798):265–9.

33.	B L, N S, J W, S Z, H S, J D, et al. An Optimized Metagenomic Approach for Virome Detection of Clinical Pharyngeal Samples With Respiratory Infection. Frontiers in microbiology [Internet]. 2020 Jul 10 [cited 2024 Jun 19];11. Available from: https://pubmed.ncbi.nlm.nih.gov/32754134/

34.	Xu Y, Lewandowski K, Jeffery K, Downs LO, Foster D, Sanderson ND, et al. Nanopore metagenomic sequencing to investigate nosocomial transmission of human metapneumovirus from a unique genetic group among haematology patients in the United Kingdom. Journal of Infection. 2020 May;80(5):571–7.

35.	Mostafa HH, Fissel JA, Fanelli B, Bergman Y, Gniazdowski V, Dadlani M, et al. Metagenomic Next-Generation Sequencing of Nasopharyngeal Specimens Collected from Confirmed and Suspect COVID-19 Patients. Leal SM, Gilligan P, editors. mBio. 2020 Dec 22;11(6):e01969-20.

36.	Charre C, Ginevra C, Sabatier M, Regue H, Destras G, Brun S, et al. Evaluation of NGS-based approaches for SARS-CoV-2 whole genome characterisation. Virus Evolution. 2020 Jul 1;6(2):veaa075.

37.	Carbo EC, Sidorov IA, Zevenhoven-Dobbe JC, Snijder EJ, Claas EC, Laros JFJ, et al. Coronavirus discovery by metagenomic sequencing: a tool for pandemic preparedness. Journal of Clinical Virology. 2020 Oct;131:104594.

38.	Van Boheemen S, Van Rijn AL, Pappas N, Carbo EC, Vorderman RHP, Sidorov I, et al. Retrospective Validation of a Metagenomic Sequencing Protocol for Combined Detection of RNA and DNA Viruses Using Respiratory Samples from Pediatric Patients. The Journal of Molecular Diagnostics. 2020 Feb;22(2):196–207.

39.	Pérez-Sautu U, Wiley MR, Iglesias-Caballero M, Pozo F, Prieto K, Chitty JA, et al. Target-independent high-throughput sequencing methods provide evidence that already known human viral pathogens play a main role in respiratory infections with unexplained etiology. Emerging Microbes & Infections. 2019 Jan;8(1):1054–65.

40.	Lewandowski K, Xu Y, Pullan ST, Lumley SF, Foster D, Sanderson N, et al. Metagenomic Nanopore Sequencing of Inﬂuenza Virus Direct from Clinical Respiratory Samples. Journal of Clinical Microbiology. 2020;58(1).

41.	Van Rijn AL, Van Boheemen S, Sidorov I, Carbo EC, Pappas N, Mei H, et al. The respiratory virome and exacerbations in patients with chronic obstructive pulmonary disease. Singanayagam A, editor. PLoS ONE. 2019 Oct 24;14(10):e0223952.

42.	Kustin T, Ling G, Sharabi S, Ram D, Friedman N, Zuckerman N, et al. A method to identify respiratory virus infections in clinical samples using next-generation sequencing. Sci Rep. 2019 Feb 22;9(1):2606.

43.	Bal A, Pichon M, Picard C, Casalegno JS, Valette M, Schuffenecker I, et al. Quality control implementation for universal characterization of DNA and RNA viruses in clinical respiratory samples using single metagenomic next-generation sequencing workflow. BMC Infect Dis. 2018 Dec;18(1):537.

44.	Madi N, Al‐Nakib W, Mustafa AS, Habibi N. Metagenomic analysis of viral diversity in respiratory samples from patients with respiratory tract infections in Kuwait. Journal of Medical Virology. 2018 Mar;90(3):412–20.

45.	Romero-Espinoza JA, Moreno-Valencia Y, Coronel-Tellez RH, Castillejos-Lopez M, Hernandez A, Dominguez A, et al. Virome and bacteriome characterization of children with pneumonia and asthma in Mexico City during winter seasons 2014 and 2015. Schildgen O, editor. PLoS ONE. 2018 Feb 15;13(2):e0192878.

46.	Sharp C, Golubchik T, Gregory WF, McNaughton AL, Gow N, Selvaratnam M, et al. Oxford Screening CSF and Respiratory samples (‘OSCAR’): results of a pilot study to screen clinical samples from a diagnostic microbiology laboratory for viruses using Illumina next generation sequencing. BMC Res Notes. 2018 Dec;11(1):120.

47.	Goya S, Valinotto LE, Tittarelli E, Rojo GL, Nabaes Jodar MS, Greninger AL, et al. An optimized methodology for whole genome sequencing of RNA respiratory viruses from nasopharyngeal aspirates. Schildgen O, editor. PLoS ONE. 2018 Jun 25;13(6):e0199714.

48.	Zhang D, Lou X, Yan H, Pan J, Mao H, Tang H, et al. Metagenomic analysis of viral nucleic acid extraction methods in respiratory clinical samples. BMC Genomics. 2018 Dec;19(1):773.

49.	Zou X, Tang G, Zhao X, Huang Y, Chen T, Lei M, et al. Simultaneous virus identification and characterization of severe unexplained pneumonia cases using a metagenomics sequencing technique. Sci China Life Sci. 2017 Mar;60(3):279–86.

50.	Greninger AL, Zerr DM, Qin X, Adler AL, Sampoleo R, Kuypers JM, et al. Rapid Metagenomic Next-Generation Sequencing during an Investigation of Hospital-Acquired Human Parainfluenza Virus 3 Infections. McAdam AJ, editor. J Clin Microbiol. 2017 Jan;55(1):177–82.

51.	Gong YN, Yang SL, Chen GW, Chen YW, Huang YC, Ning HC, et al. A metagenomics study for the identification of respiratory viruses in mixed clinical specimens: an application of the iterative mapping approach. Arch Virol. 2017 Jul;162(7):2003–12.

52.	Lewandowska DW, Schreiber PW, Schuurmans MM, Ruehe B, Zagordi O, Bayard C, et al. Metagenomic sequencing complements routine diagnostics in identifying viral pathogens in lung transplant recipients with unknown etiology of respiratory infection. Schildgen O, editor. PLoS ONE. 2017 May 23;12(5):e0177340.

53.	Cui L, Wu B, Zhu X, Guo X, Ge Y, Zhao K, et al. Identification and genetic characterization of a novel circular single-stranded DNA virus in a human upper respiratory tract sample. Arch Virol. 2017 Nov;162(11):3305–12.

54.	Wang Y, Zhu N, Li Y, Lu R, Wang H, Liu G, et al. Metagenomic analysis of viral genetic diversity in respiratory samples from children with severe acute respiratory infection in China. Clinical Microbiology and Infection. 2016 May;22(5):458.e1-458.e9.

55.	Graf EH, Simmon KE, Tardif KD, Hymas W, Flygare S, Eilbeck K, et al. Unbiased Detection of Respiratory Viruses by Use of RNA Sequencing-Based Metagenomics: a Systematic Comparison to a Commercial PCR Panel. Caliendo AM, editor. J Clin Microbiol. 2016 Apr;54(4):1000–7.

56.	Thorburn F, Bennett S, Modha S, Murdoch D, Gunson R, Murcia PR. The use of next generation sequencing in the diagnosis and typing of respiratory infections. Journal of Clinical Virology. 2015 Aug;69:96–100.

57.	Zoll J, Rahamat-Langendoen J, Ahout I, Jonge MI de, Jans J, Huijnen MA, et al. Direct multiplexed whole genome sequencing of respiratory tract samples reveals full viral genomic information. Journal of Clinical Virology. 2015 May;66:6–11.

58.	Moesker FM, Van Kampen JJA, Van Der Eijk AA, Van Rossum AMC, De Hoog M, Schutten M, et al. Human bocavirus infection as a cause of severe acute respiratory tract infection in children. Clinical Microbiology and Infection. 2015 Oct;21(10):964.e1-964.e8.

59.	Fischer N, Indenbirken D, Meyer T, Lütgehetmann M, Lellek H, Spohn M, et al. Evaluation of Unbiased Next-Generation Sequencing of RNA (RNA-seq) as a Diagnostic Method in Influenza Virus-Positive Respiratory Samples. Tang YW, editor. J Clin Microbiol. 2015 Jul;53(7):2238–50.

60.	Huang W, Wang G, Zhuge J, Nolan SM, Dimitrova N, Fallon JT. Whole-Genome Sequence Analysis Reveals the Enterovirus D68 Isolates during the United States 2014 Outbreak Mainly Belong to a Novel Clade. Sci Rep. 2015 Oct 15;5(1):15223.

61.	Young JC, Chehoud C, Bittinger K, Bailey A, Diamond JM, Cantu E, et al. Viral Metagenomics Reveal Blooms of Anelloviruses in the Respiratory Tract of Lung Transplant Recipients. American Journal of Transplantation. 2015 Feb;15(1):200–9.

62.	Taboada B, Espinoza MA, Isa P, Aponte FE, Arias-Ortiz MA, Monge-Martínez J, et al. Is There Still Room for Novel Viral Pathogens in Pediatric Respiratory Tract Infections? Kapoor A, editor. PLoS ONE. 2014 Nov 20;9(11):e113570.

63.	Alquezar-Planas DE, Mourier T, Bruhn CAW, Hansen AJ, Vitcetz SN, Mørk S, et al. Discovery of a divergent HPIV4 from respiratory secretions using second and third generation metagenomic sequencing. Sci Rep. 2013 Sep 2;3(1):2468.

64.	Lysholm F, Wetterbom A, Lindau C, Darban H, Bjerkner A, Fahlander K, et al. Characterization of the Viral Microbiome in Patients with Severe Lower Respiratory Tract Infections, Using Metagenomic Sequencing. PLoS One. 2012 Feb 15;7(2):e30875.

65.	Yang J, Yang F, Ren L, Xiong Z, Wu Z, Dong J, et al. Unbiased Parallel Detection of Viral Pathogens in Clinical Samples by Use of a Metagenomic Approach. J Clin Microbiol. 2011 Oct;49(10):3463–9.

66.	De Vries M, Deijs M, Canuti M, Van Schaik BDC, Faria NR, Van De Garde MDB, et al. A Sensitive Assay for Virus Discovery in Respiratory Clinical Samples. Poon LLM, editor. PLoS ONE. 2011 Jan 24;6(1):e16118.
